# Quercetin attenuates skin inflammation and fibrosis in systemic sclerosis by targeting the RELA/c-Jun axis to suppress th17 cell responses

**DOI:** 10.3389/fimmu.2026.1863530

**Published:** 2026-06-03

**Authors:** Xiangzhen Kong, Hiochon Leong, Min Du, Jingyi Yang, Jingzhen Lai, Ziying Dong, Menghua Hu, Yifei Gu, Yexin Li, Qiaorui Tan, Haiyan Chu, Qingmei Liu, Yanyun Ma, Weihong Xu, Shanji Li, Yuxiao Chen, Dongdong Chen, Yuanyuan Chen, Wei Lu, Jie Zhu, Jiucun Wang, Lei Wang

**Affiliations:** 1 Division of Rheumatology, Shanghai TCM-Integrated Hospital, Shanghai University of Traditional Chinese Medicine, Shanghai, China; 2 Human Phenome Institute, Fudan University, Shanghai, China; 3 State Key Laboratory of Genetics and Development of Complex Phenotypes, Collaborative Innovation Center for Genetics and Development, School of Life Sciences, Fudan University, Shanghai, China; 4 Department of Dermatology, Huashan Hospital, Fudan University, Shanghai, China; 5 Department of Pathology, Huadong Hospital, Fudan University, Shanghai, China; 6 Department of Plastic and Aesthetic Surgery, The Second Affiliated Hospital of Soochow University, Suzhou, Jiangsu, China; 7 Faculty of Applied Science, University of British Columbia, Vancouver, BC, Canada; 8 Research Unit of Dissecting the Population Genetics and Developing New Technologies for Treatment and Prevention of Skin Phenotypes and Dermatological Diseases, Chinese Academy of Medical Sciences, Peking Union Medical College, Peking Union Medical College Hospital, Beijing, China; 9 Academy for Engineering & Technology, Fudan University, Shanghai, China; 10 Department of Clinical Laboratory, Shibei Hospital, Jing’an District, Shanghai, China; 11 Department of Obstetrics and Gynecology, Shanghai General Hospital, Shanghai Jiao Tong University School of Medicine, Shanghai, China; 12 Department of Stomatology, Hangzhou First People’s Hospital, Hangzhou, China; 13 Anesthesiology department of Shanghai Putuo Maternity and Infant Health Hospital, Shanghai, China

**Keywords:** c-jun, IL-17 signaling, quercetin, RelA (p65), skin fibrosis, systemic sclerosis, Th17 cells

## Abstract

**Background:**

Aberrant Th17 cell activation and interleukin-17 (IL-17) production drive skin inflammation and progressive fibrosis in systemic sclerosis (SSc). Identifying small molecules that can precisely modulate these pathogenic T cell responses is crucial for developing novel SSc therapies.

**Methods:**

We utilized primary CD4^+^ T cells isolated from SSc patients and a bleomycin-induced SSc mouse model to evaluate the immunomodulatory effects of quercetin. Direct molecular target engagement was rigorously validated using Drug Affinity Responsive Target Stability (DARTS) and Cellular Thermal Shift Assay (CETSA).

**Results:**

In patient-derived CD4^+^ T cells, quercetin treatment significantly suppressed the secretion of pro-inflammatory cytokines, including IL-17A and IFN-γ. Mechanistically, we demonstrated through DARTS and CETSA that quercetin directly binds to and stabilizes transcription factors RELA (p65) and c-Jun. This interaction effectively inhibited their phosphorylation and subsequent nuclear translocation, thereby dampening the IL-17 signaling cascade. *In vivo*, oral administration of quercetin significantly attenuated skin thickness, collagen deposition, and myofibroblast activation in SSc mice.

**Conclusion:**

Our findings identify the RELA/c-Jun axis as a critical target for modulating T cell pathogenicity in SSc and suggest that quercetin holds therapeutic potential as an immunometabolic modulator to alleviate skin fibrosis.

## 1 Introduction

Systemic sclerosis (SSc) is a chronic autoimmune fibrotic disorder characterized by excessive extracellular matrix deposition and profound vascular dysfunction, leading to severe, irreversible organ damage for which curative treatments are currently unavailable (1). Despite its relatively low prevalence, the substantial socioeconomic burden SSc imposes on patients and healthcare systems highlights the urgent need for novel interventions (2). The pathogenesis of SSc is highly multifactorial, with immune dysregulation acting as a central catalyst. Activated T helper cells aberrantly infiltrate the dermis, secreting a cascade of pro-inflammatory cytokines—notably IL-17A, IFN-γ, and IL-4—which synergistically drive sustained inflammation and provoke profibrotic responses (3–5). Increasing evidence emphasizes the critical involvement of the IL-17/Th17 axis in SSc-associated pathology (4, 5). Elevated serum IL-17A levels in SSc patients strongly correlate with upregulated Th17-associated cytokine profiles, extensive cutaneous involvement, and an increased incidence of pulmonary arterial hypertension (6). Mechanistically, IL-17 and Th17 cells not only induce the production of downstream pro-inflammatory mediators but also activate critical signaling hubs, such as the NF-κB and MAPK pathways, thereby amplifying the immune cascade and accelerating fibrogenesis (7, 8). Consequently, therapeutically targeting the IL-17/Th17 axis presents a promising strategy for restoring immune homeostasis and halting fibrosis in SSc. Currently, clinical management focuses primarily on alleviating organ-specific complications such as interstitial lung disease, pulmonary arterial hypertension, and skin fibrosis (9, 10). Immunosuppressive agents, including mycophenolate mofetil and cyclophosphamide, are commonly used to slow disease progression. Recently, targeted therapies such as nintedanib and tocilizumab have shown efficacy in stabilizing lung function and are now approved for SSc-associated interstitial lung disease (11). Emerging options, including rituximab, romilkimab, mesenchymal stem cells, and CAR-T cell therapy, demonstrate potential in ameliorating fibrosis and modulating immune responses (12, 13). However, these approaches are often associated with significant adverse effects, and concerns remain regarding long-term efficacy and safety of CAR-T therapy, including risks of relapse, cytokine release syndrome, and neurotoxicity. Thus, effective and well-tolerated immunomodulatory therapies for SSc are still urgently needed.

In this context, identifying small-molecule immunomodulators possessing favorable safety profiles has garnered significant attention as complementary or alternative therapeutics (14, 15). Among these, naturally occurring flavonoids, particularly quercetin (a core bioactive constituent of the traditional herb Astragalus), exhibit potent immunomodulatory and antifibrotic activities, demonstrating broad therapeutic potential across various autoimmune diseases (16, 17). Recent studies have demonstrated that quercetin can induce cellular senescence in follicular helper T (Tfh) cells and suppress the production of inflammatory cytokines, thereby alleviating disease progression in systemic lupus erythematosus (18). Furthermore, quercetin has been shown to inhibit the activation and differentiation of Th2 cells, effectively mitigating airway hyperresponsiveness and allergic inflammation (19).

Preclinical evidence indicates that Astragalus and its key components (e.g., astragaloside IV, quercetin) can significantly attenuate collagen and fibronectin accumulation in bleomycin-induced SSc models, predominantly via the suppression of the TGF-β/Smad3 cascade (20). Nevertheless, the precise intracellular targets of quercetin within pathogenic T cells, and its ability to decouple T-cell activation from downstream fibrotic responses in SSc, remain incompletely defined. Autoimmune imbalance triggers the onset of inflammation and fibrosis in SSc; therefore, modulating the SSc immune system may inhibit the production of pro-fibrotic drivers at their source, thereby suppressing fibrogenesis (21). To date, however, the impact of quercetin on skin fibrosis in SSc has not been investigated, and its specific effects and underlying mechanisms within the aberrant T-cell populations of SSc patients remain unknown. Notably, the IL-17 signaling pathway plays a pivotal role in promoting skin fibrosis in SSc (22). Furthermore, flavonoids are recognized for their potent antioxidant properties, with quercetin being the most extensively studied due to its low toxicity and superior radical-scavenging activity (23, 24). Quercetin undergoes metabolism across multiple organs, and its metabolites retain potent antioxidant effects that significantly surpass those of other flavonoids (25). Given that SSc is a multi-systemic disorder involving various organs, quercetin is uniquely positioned to address the multi-organ imbalances characteristic of the disease. Consequently, investigating the effects of quercetin on skin inflammation and fibrosis in SSc is of great significance for the development of novel therapeutic strategies.

This study aimed to systematically dissect the immunomodulatory mechanisms of quercetin in SSc. By employing an integrated approach that utilized primary CD4^+^ T cells isolated from SSc patients, rigorous target-engagement assays (including DARTS and CETSA), and robust *in vitro* and *in vivo* experimental validations, we sought to elucidate the molecular targets, and immunological pathways mediating the anti-inflammatory and anti-fibrotic effects of this small molecule. Building upon initial molecular docking, we further performed molecular dynamics simulations to analyze the interaction between quercetin and its targets. The binding affinity between quercetin and these proteins was subsequently validated through *in vitro* assays, ensuring high precision in target identification. Our findings reveal that quercetin substantially mitigates skin inflammation and fibrosis in SSc by directly binding to RELA (p65) and c-Jun, effectively modulating the IL-17 signaling cascade, with the suppression of NF-κB and c-Jun phosphorylation serving as critical regulatory checkpoints.

## 2 Materials and methods

### 2.1 Ethics statement and human sample collection

All human and animal studies were conducted in accordance with the Declaration of Helsinki and approved by the Ethics Committee of Shanghai Integrated Traditional Chinese and Western Medicine Hospital Affiliated to Shanghai University of Traditional Chinese Medicine (Approval No. 2024-006-1). Written informed consent was obtained from all participating systemic sclerosis (SSc) patients. Animal experiments were approved by the Institutional Animal Care and Use Committee Fudan university (Approval No.2024-MS-D-31).

### 2.2 Bioinformatics screening and molecular docking

Molecular targets associated with the 20 active compounds were obtained, yielding 210 candidate targets. To identify SSc-related targets, data was sourced from GeneCards (https://www.genecards.org/) (relevance score > 10), DisGeNET (https://disgenet.com/), and the Therapeutic Target Database (https://idrblab.net/ttd/), resulting in 3202 potential targets. Common targets between Astragalus compounds and SSc were identified using Venny 2.1 (https://bioinfogp.cnb.csic.es/tools/venny/) and visualized using Cytoscape v3.10.1.

Three-dimensional structures of the active compounds were retrieved from the PubChem database (https://pubchem.ncbi.nlm.nih.gov/) and optimized using Chem3D (version 23.0.1). Target protein structures were obtained from the Protein Data Bank (https://www.rcsb.org/) and pre-processed in PyMOL (version 3.1) to remove water molecules and bound ligands. Hydrogen atoms were added to protein structures in AutoDockTools (version 1.5.7). Docking simulations were performed using AutoDock Vina (version 1.1.2), and the binding interactions were visualized in PyMOL. Binding modes were analyzed to evaluate interaction sites and subunits involved in the ligand–target protein complexes.

### 2.3 Bleomycin-Induced SSc mouse model and treatment

Specific pathogen-free (SPF), 8-week-old C57BL/6J mice were housed in the SPF animal facility at the School of Life Sciences, Fudan University. All mice were maintained in standard individually ventilated cages (IVC) under controlled environmental conditions (12/12 h light/dark cycle, temperature 22 ± 2 °C, and humidity 50 ± 10%), with ad libitum access to standard rodent chow and autoclaved water. Daily animal husbandry, including cage separation, bedding replacement, and water changing, was strictly performed by dedicated professional staff in accordance with SPF requirements to minimize animal stress and suffering.

The dorsal fur of the mice was shaved using an electric clipper. After two days of acclimatization, skin fibrosis was induced by subcutaneous injection of sterile bleomycin (1 mg/mL in PBS, 100 μL) into a 1 cm² dorsal region. To minimize pain and distress during the painful subcutaneous injection, mice were anesthetized using an isoflurane anesthesia machine (induced with 3-4% isoflurane and maintained with 1.5-2% isoflurane in oxygen). Three days post-bleomycin injection, the mice were orally administered Astragalus extract or quercetin at the designed doses daily via gavage (gavage volume ≤ 200 μL, Astragalus extract 10 mg/kg/day; Quercetin: 50 mg/kg/day). Oral gavage was performed by trained personnel using proper restraint techniques on conscious mice without the need for anesthesia.

Four weeks after bleomycin injection, all mice were humanely euthanized via an overdose of inhaled isoflurane (prolonged exposure to 5% isoflurane until breathing ceased), followed by cervical dislocation to ensure death. Subsequently, skin tissues and spleens were harvested. The skin samples were fixed and subjected to hematoxylin-eosin (H&E), Masson’s trichrome, and immunohistochemical staining. Spleens were homogenized, stained with flow cytometry antibodies, and analyzed to assess immune cell population changes.

### 2.4 Isolation of primary SSc CD4^+^ T cells and dermal fibroblasts

Peripheral venous blood (10 mL) was collected from SSc patients. Following plasma collection, peripheral blood mononuclear cells (PBMCs) were isolated utilizing a Ficoll density gradient separation solution. CD4^+^ T cells were positively selected from the PBMC fraction using human CD4^+^ magnetic microbeads and a magnetic sorting column according to the manufacturer’s instructions. Primary SSc dermal fibroblasts were isolated employing the tissue explant method. Briefly, acquired SSc skin tissues were minced into ~0.3 mm³ fragments and cultured under sterile coverslips in DMEM supplemented with 10% FBS. Upon observing robust fibroblast outgrowth (2–3 days), tissue fragments were removed, and fibroblasts were propagated for downstream assays.

### 2.5 Target engagement assays (DARTS and CETSA)

To validate the physical binding between quercetin and its intracellular targets, Drug Affinity Responsive Target Stability (DARTS) and Cellular Thermal Shift Assays (CETSA) were performed. For DARTS, total cellular proteins were extracted using a non-denaturing lysis buffer, and protein concentrations were subsequently determined. The cell lysates were partitioned into designated groups and incubated with either quercetin or an equivalent volume of vehicle control. For the proteolytic digestion, Pronase was added to each sample at a final concentration of 100 μg/mL and incubated at room temperature for 30 min. The reaction was terminated by the addition of a protease inhibitor cocktail. Subsequently, samples were supplemented with protein loading buffer, denatured at 100 °C for 10 min, and subjected to Western blot analysis. For CETSA, Jurkat T cells were incubated with either DMSO or quercetin (16 μM) for 1 h. Cells were then aliquoted and subjected to a temperature gradient (55, 60, 65, 70, and 77 °C) for 5 min, followed by three rapid freeze-thaw cycles in liquid nitrogen after the addition of RIPA buffer. Soluble protein fractions were isolated by centrifugation and subjected to Western blotting to evaluate the thermal stabilization of the target proteins.

### 2.6 T cell-fibroblast co-culture system

Purified SSc CD4^+^ T cells were cultured in RPMI-1640 medium supplemented with filtered SSc patient plasma (1:1 ratio) to mimic the disease microenvironment. Cells were treated with either the botanical extract (500 μg/mL) or purified quercetin (16 μM) for 24 h. The optimal concentrations of the botanical extract and quercetin for cell treatment were screened using the CCK-8 assay (**Supplementary Figure 1**). Post-treatment, the pre-conditioned CD4^+^ T cells were transferred into the upper chamber of a Transwell co-culture system (0.4 μm pore size), while primary SSc dermal fibroblasts were seeded in the lower chamber. Following a 24 h co-culture, fibroblasts were harvested using RIPA lysis buffer for subsequent immunoblotting analysis of fibrotic markers.

### 2.7 Flow cytometry analysis

After cervical dislocation, the mouse spleen was collected. In a biosafety cabinet, the spleen was washed with PBS and then treated with antibiotics (double-antibiotic solution), followed by another PBS rinse. The spleen was placed in a 70 μm cell strainer and gently ground using a syringe plunger. The strainer was rinsed with 2 mL of PBS, and the filtrate was collected into a 15 mL centrifuge tube. The suspension was centrifuged at 1500 rpm for 5 minutes, and the supernatant was discarded. The cell pellet was resuspended in 3 mL of PBS for washing. Next, 5 mL of red blood cell lysis buffer was added, and the mixture was incubated on ice for 10 minutes before centrifugation at 1500 rpm for 5 minutes. The cells were washed once more with 3 mL of PBS. The cell pellet was resuspended in RPMI-1640 medium supplemented with 10% FBS and transferred to a 24-well plate for culture. T cells were stimulated for 4 hours with Phorbol 12-myristate 13-acetate (PMA), ionomycin, and brefeldin A. After stimulation, the cells were collected and centrifuged at 1500 rpm for 5 minutes, followed by a PBS wash. For staining, 100 μL of PBS containing 1% FBS was used to resuspend the cells, followed by the addition of FVS700 viability dye, anti-mouse CD45-PE, anti-mouse CD3-FITC, anti-mouse CD4-BV510, anti-mouse IL-4-BV421, anti-mouse IL-17A-CF594, and anti-mouse IFN-γ-APC antibodies. The cells were incubated at room temperature for 30 minutes in the dark, then centrifuged at 1500 rpm for 5 minutes and washed once with 3 mL of PBS containing 1% FBS. Next, fixation and permeabilization buffer was added, and the cells were incubated at room temperature for 20 minutes. After permeabilization, 100 μL of PBS containing 1% FBS and intracellular antibodies (anti-mouse IL-4-BV421, anti-mouse IL-17A-CF594, and anti-mouse IFN-γ-APC) was added, followed by a 30-minute incubation in the dark. The cells were centrifuged at 1500 rpm for 3 minutes and washed once with PBS containing 1% FBS. Finally, the cells were resuspended in 400 μL of PBS with 1% FBS and subjected to flow cytometry analysis. The flow cytometry gating strategy for CD4^+^ T cells is shown in **Supplementary Figure 2**.

### 2.8 RNA extraction, qPCR, and western blotting

Cells were lysed using TRIzol reagent (Catalog No. 15596018, Thermo Fisher) for a duration of 15 minutes. Following the addition of chloroform, the lysate was incubated at room temperature for 5 minutes. The mixture was then subjected to centrifugation at 13,800 × g for 15 minutes. The upper phase, containing RNA, was carefully aspirated and precipitated with an equal volume of isopropanol. The RNA was subsequently washed with 75% ethanol and dissolved in DEPC-treated water. RNA concentration was quantified using a NanoDrop 2000 spectrophotometer. For cDNA synthesis, 2 μg of RNA was reverse transcribed using the Hifair^®^ II First-Strand cDNA Synthesis SuperMix (Catalog No. 11141ES60, Yeasen) in accordance with the manufacturer’s protocol. The resulting cDNA was then analyzed via qPCR employing the Hieff UNICON^®^ Universal Blue qPCR SYBR Green Master Mix (Catalog No. 11184ES25, Yeasen), following the manufacturer’s guidelines. Primer sequences for qPCR are shown in **Supplementary Table 1**.

Approximately 1 × 10^7^ cells were washed with PBS, and total intracellular proteins were extracted using ice-cold RIPA lysis buffer (Beyotime, P0013) supplemented with protease and phosphatase inhibitor cocktails for 10 min on ice. Insoluble debris was pelleted by centrifugation at 13,800 rpm for 15 min at 4 °C, and protein concentrations were determined utilizing a BCA protein assay kit. Equal amounts of protein samples were mixed with 4× loading buffer, boiled for 10 min, separated by 10% SDS-PAGE, and electrophoretically transferred onto polyvinylidene difluoride (PVDF) membranes (Millipore, Billerica, MA, USA). Membranes were blocked with 5% bovine serum albumin (BSA; Servicebio, China) and incubated overnight at 4 °C with primary antibodies targeting GAPDH, NF-κB p65, Phospho-NF-κB p65, c-JUN, and Phospho-c-JUN (all diluted 1:1000, Cell Signaling Technology, USA). Following primary incubation, membranes were probed with HRP-conjugated anti-rabbit secondary antibodies (Servicebio, China) for 1 h at room temperature. Protein bands were visualized and densitometrically quantified using Image-QuantTL software (General Electric Company, USA).

### 2.9 Statistical analysis

Data are presented as mean ± standard deviation (SD). The exact sample size (n) for each experimental group is indicated in the respective figure legends. For comparisons between two independent groups, an unpaired Student’s t-test was employed. For multiple group comparisons involving a single variable, one-way analysis of variance (ANOVA) followed by Tukey’s or Dunnett’s *post-hoc* test was performed. For experiments involving two variables, two-way ANOVA with Sidak’s multiple comparisons test was used. All statistical analyses were conducted using GraphPad Prism software (version 9.5.1, La Jolla, CA, USA). Normality and homogeneity of variance were assessed prior to parametric testing. A p-value < 0.05 was considered statistically significant. Significance levels are denoted as: * p < 0.05, ** p < 0.01, *** p < 0.001, and **** p < 0.0001.

## 3 Results

### 3.1 Initial bioinformatics screening identifies the IL-17/Th17 pathway as a primary immunotherapeutic target

**Figure 1:**
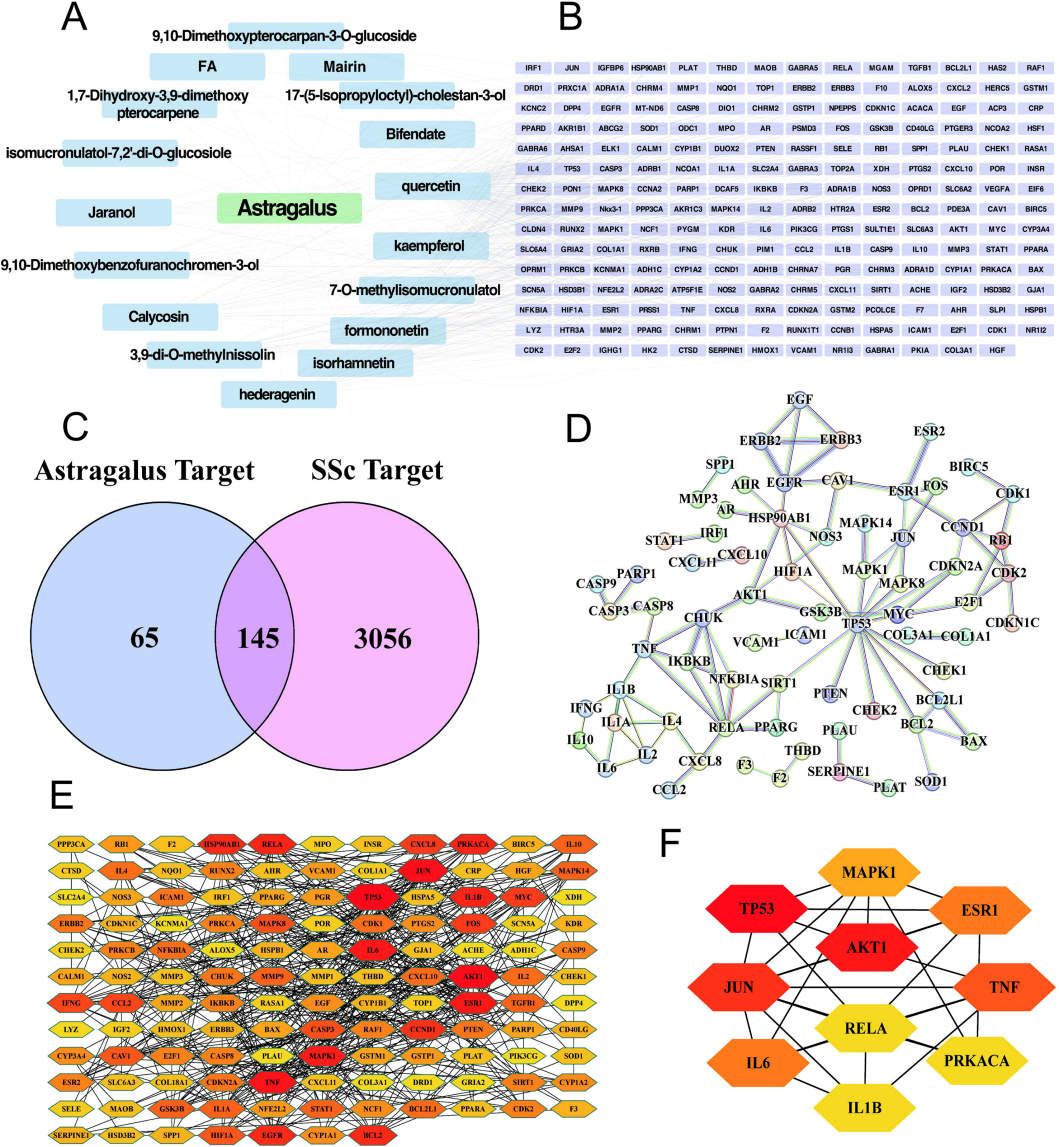
Identification of bioactive compounds and therapeutic targets in SSc. **(A, B)** Network of 17 active compounds and their 209 predicted biological targets. **(C)** Venn diagram identifying 145 shared targets between compound-related and SSc-associated genes. **(D)** PPI network of the overlapping targets. **(E, F)** Topological analysis and identification of the top 10 hub genes ranked by degree score.

Utilizing computational screening databases, we retrieved 17 active compounds from the botanical source, which mapped to 209 distinct molecular targets (**Figures 1A, B**). Intersecting these targets with 3,202 SSc-associated genes identified a core set of 145 potential therapeutic targets (**Figure 1C**). To delineate the central regulatory nodes, a protein-protein interaction (PPI) network was constructed (**Figure 1D**). Topological analysis revealed that the top 10 proteins by degree were TP53, AKT1, JUN, TNF, ESR1, IL6, MAPK1, PRKACA, IL1B, and RELA (**Figures 1E, F**).

**Figure 2:**
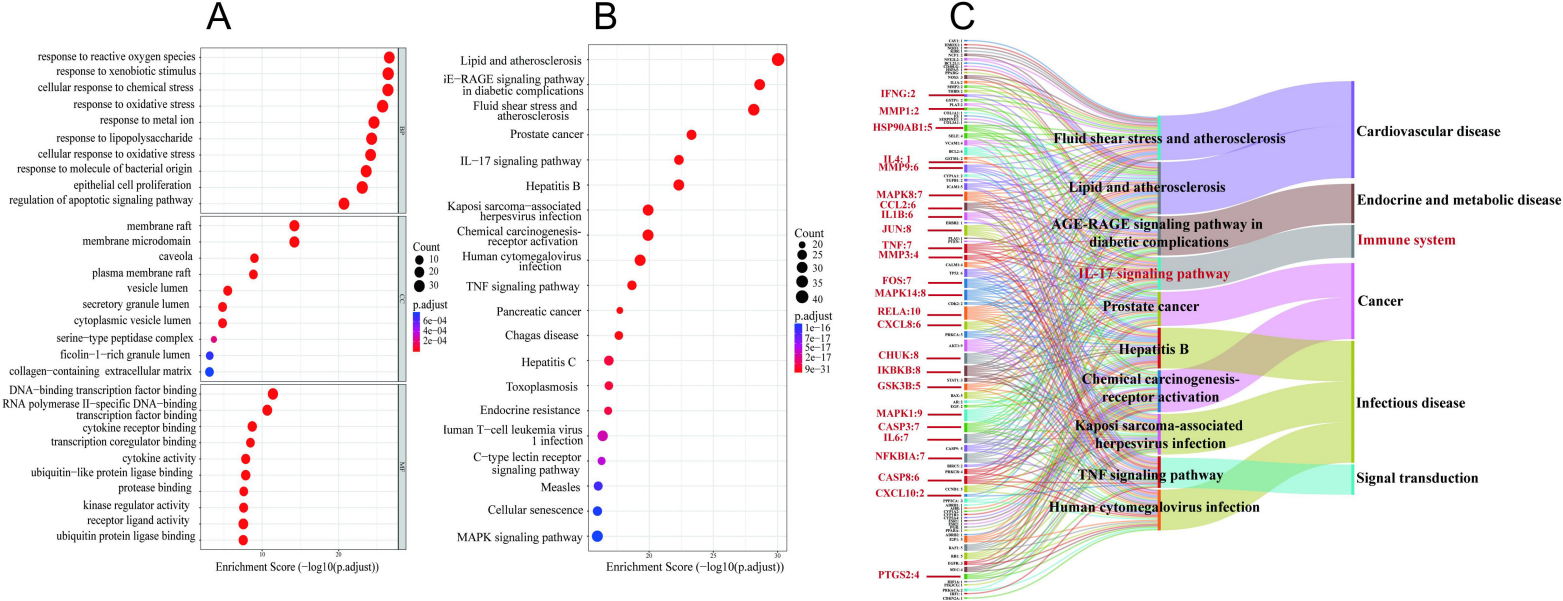
Functional enrichment and pathway analysis of common targets. **(A)** GO enrichment analysis across biological processes (BP), cellular components (CC), and molecular functions (MF). **(B)** KEGG pathway enrichment analysis. **(C)** Sankey diagram illustrating the interplay among key genes, biological functions, and major signaling pathways (e.g., IL-17 pathway).

GO enrichment analysis of the 145 candidate targets revealed primary involvement in biological processes related to response to reactive oxygen species, xenobiotic stimuli, and cellular response to chemical stress. Cellular component analysis showed enrichment in extrinsic components of the inner leaflet of the plasma membrane. Molecular function analysis indicated enrichment in DNA-binding transcription factor binding, RNA polymerase II-specific transcription factor binding, and transcriptional coregulator interactions (**Figure 2A**). Crucially, KEGG pathway enrichment analysis identified a highly significant enrichment in the IL-17 signaling pathway (**Figure 2B**). A Sankey diagram further illustrated that 25 genes were intimately enriched in this pathway, which is closely associated with immune response (**Figure 2C**). Collectively, these predictive findings strongly suggest that the active constituents exert their immunomodulatory effects primarily by targeting the pathogenic IL-17 network.

### 3.2 *In Vivo* efficacy: the botanical source of quercetin attenuates fibrosis by modulating pathogenic Th1/Th17 subsets

**Figure 3:**
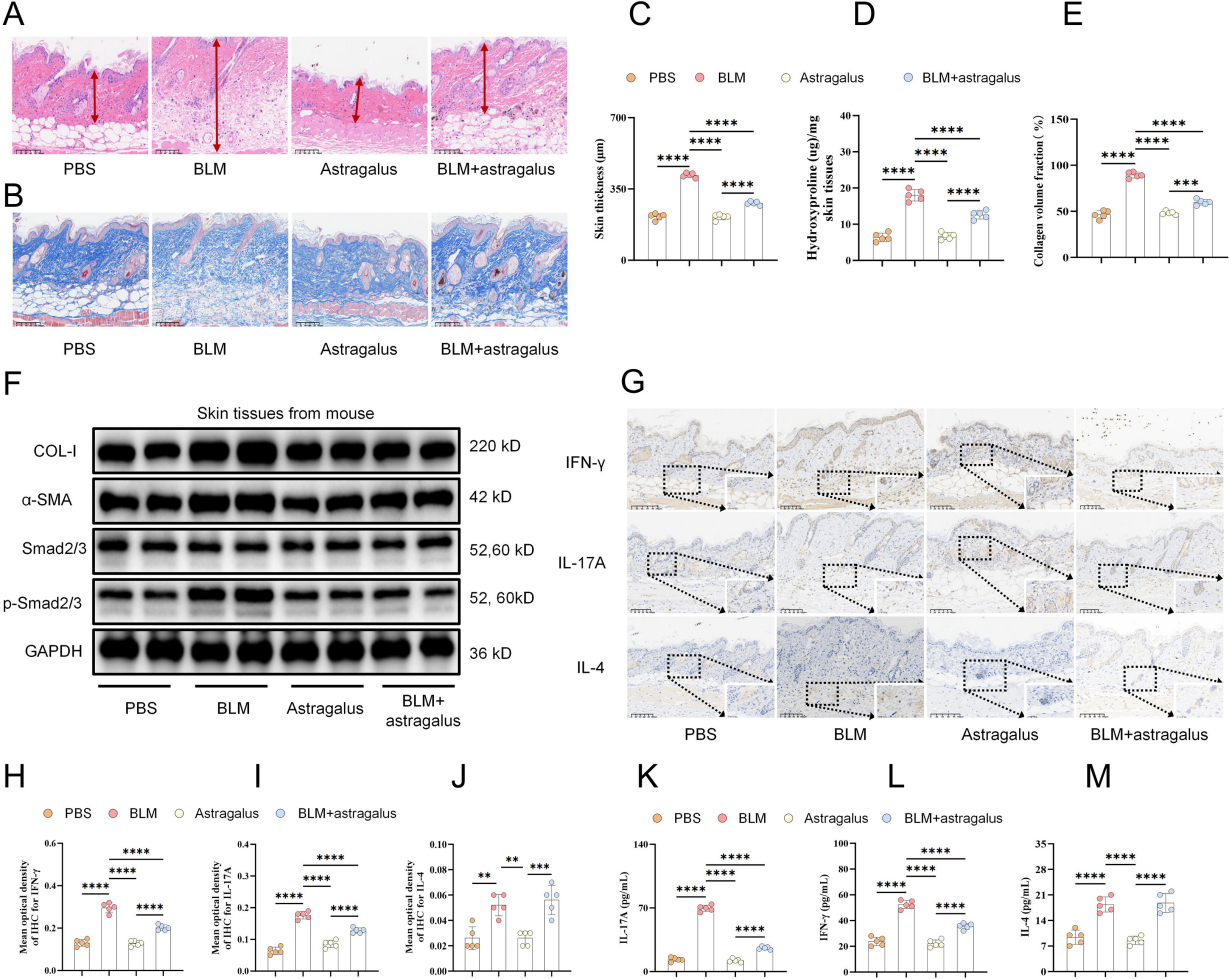
Astragalus extract alleviates skin fibrosis and inflammation in BLM-induced SSc mice. **(A–E)** Representative H&E and Masson’s trichrome staining, with quantification of dermal thickness, hydroxyproline content, and collagen volume fraction. Scale bar = 100 μm. **(F)** Western blot analysis of COL-I, α-SMA, and Smad2/3 signaling. **(G–J)** Representative IHC images and IOD quantification of dermal IFN-γ, IL-17A, and IL-4. **(K–M)** ELISA quantification of plasma IL-17A, IFN-γ, and IL-4. Data are mean ± SD; **p < 0.01, ***p < 0.001, ****p < 0.0001 (one-way ANOVA).

To evaluate the therapeutic potential of the compound-rich botanical extract in skin fibrosis, bleomycin (BLM)-induced fibrotic mice were treated by oral gavage daily for 25 days. H&E (**Figure 3A**) and Masson’s trichrome staining (**Figure 3B**) demonstrated that this treatment robustly attenuated skin thickness (**Figure 3C**) and significantly reduced both hydroxyproline (**Figure 3D**) and collagen content (**Figure 3E**) compared to the BLM vehicle group (p < 0.05). Western blot analysis further corroborated these findings, showing marked downregulation of profibrotic proteins, including COL-I, α-SMA, Smad2/3, and p-Smad2/3 (**Figure 3F**; **Supplementary Figure 3**). Consistent with our bioinformatics predictions, immunohistochemistry revealed a striking reduction in IFN-γ and IL-17A expression within the dermis, suggesting modulation via the IL-17 signaling pathway (**Figures 3G–J**). ELISA of mouse plasma further confirmed that the treatment suppressed BLM-induced systemic IL-17A and IFN-γ levels, while IL-4 remained unchanged (**Figures 3K–M**).

**Figure 4:**
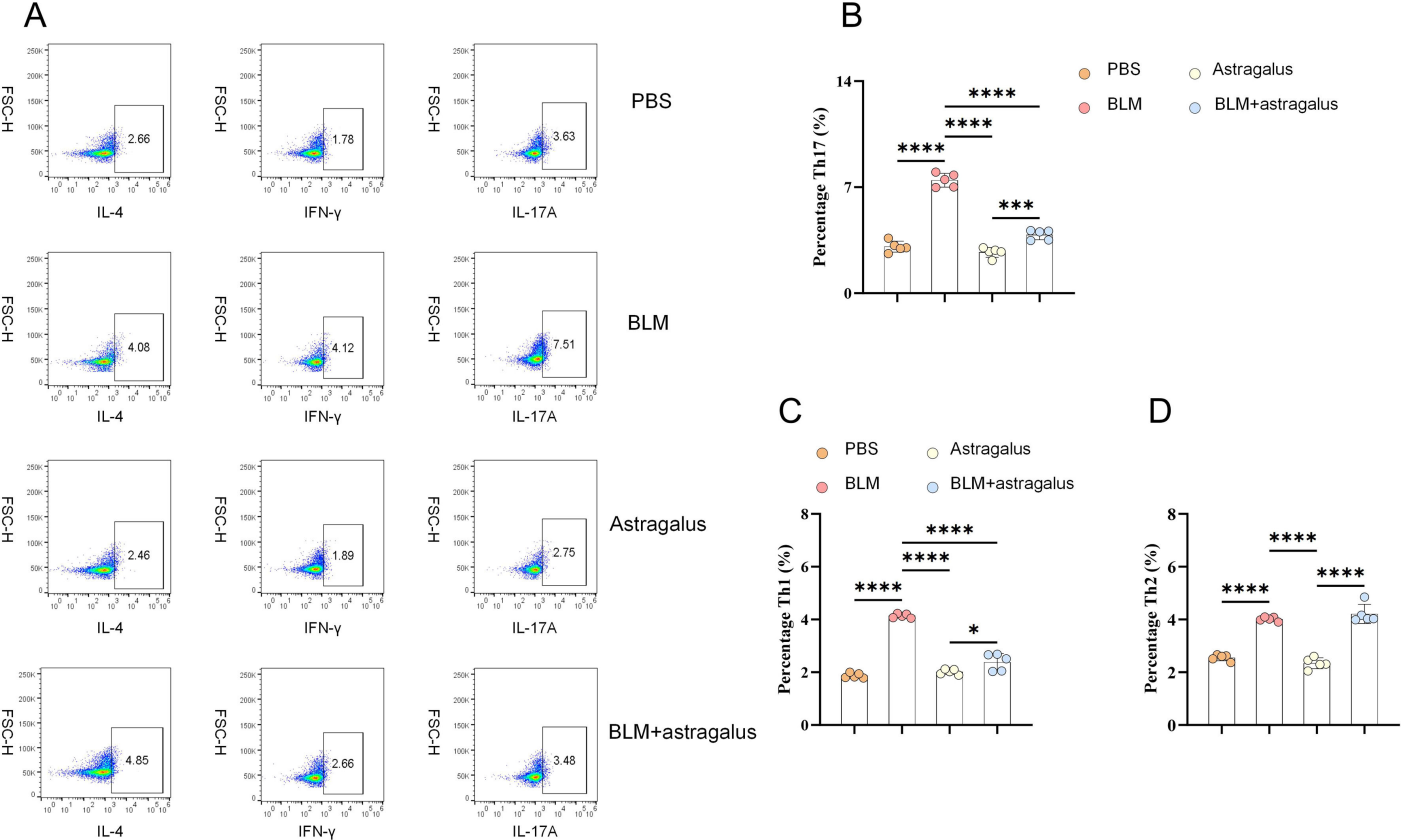
Astragalus extract inhibits Th1 and Th17 cell differentiation in BLM-induced SSc mice. **(A)** Representative flow cytometry plots of Th1 (IFN-γ^+^), Th17 (IL-17A^+^), and Th2 (IL-4^+^) subsets gated from splenic CD4^+^ T cells. **(B–D)** Statistical percentages of Th17 **(B)**, Th1 **(C)**, and Th2 **(D)** cells across treatment groups. Data are mean ± SD; *p < 0.05, ***p < 0.001, ****p < 0.0001 (one-way ANOVA).

To decipher the systemic immunological shifts, spleens from BLM-induced mice were analyzed by flow cytometry. The treatment significantly impaired the differentiation of pathogenic Th1 and Th17 subsets (by 1.46% and 4.03%, respectively, p < 0.05), without altering Th2 populations (**Figures 4A–D**). The gating strategy for CD4^+^ T cells is detailed in **Supplementary Figure 1**.

### 3.3 Target-engagement assays (DARTS/CETSA) provide definitive evidence of quercetin binding to the RELA/c-Jun complex

**Figure 5:**
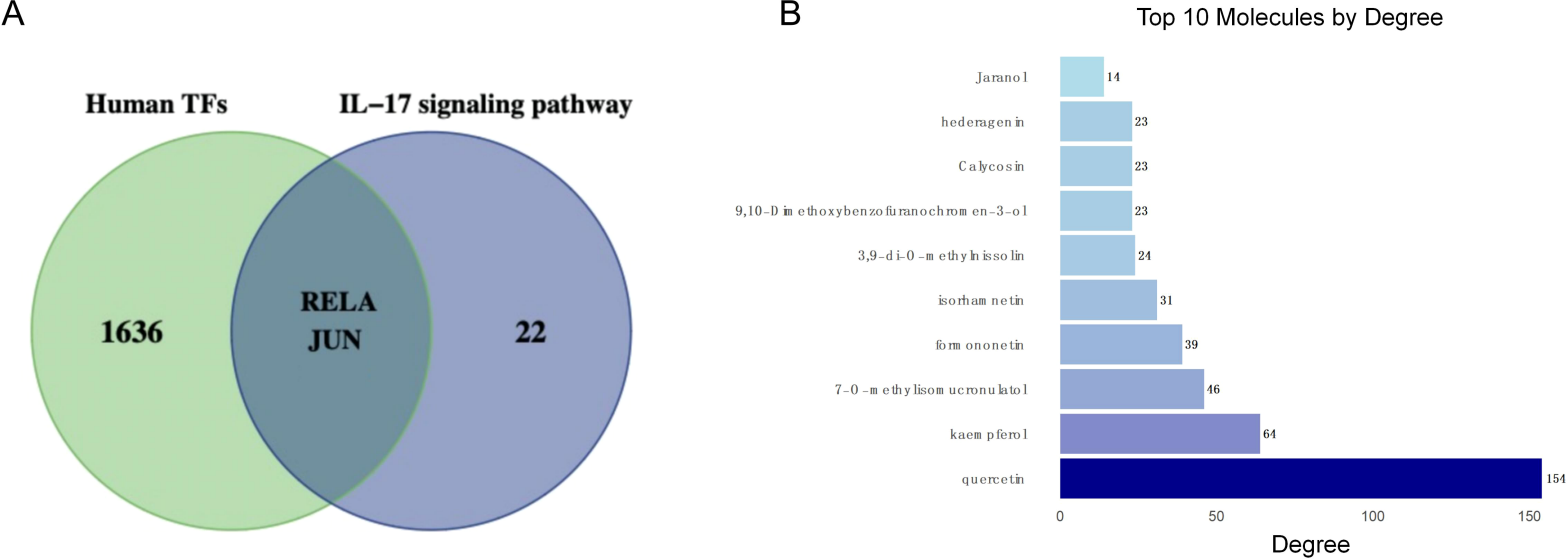
Identification of core transcription factors and bioactive compounds targeting IL-17 signaling. **(A)** Venn diagram illustrating the intersection of IL-17 pathway genes and human transcription factors (TFs), identifying RELA and JUN as key nodes. **(B)** Top 10 active compounds ranked by degree score, with quercetin identified as the primary constituent.

Having established the IL-17 signaling pathway as a key mechanism, we focused on the specific intracellular regulators. Transcription factors play a pivotal role in this pathway by regulating IL-17A and IFN-γ expression (26–28). To further elucidate how small molecules modulate this cascade, we cross-referenced IL-17 pathway-associated genes with human transcription factors, highlighting JUN and RELA as critical regulatory targets (**Figure 5A**). Additionally, topological analysis identified quercetin as the most highly connected active compound within the extract (**Figure 5B**).

**Figure 6:**
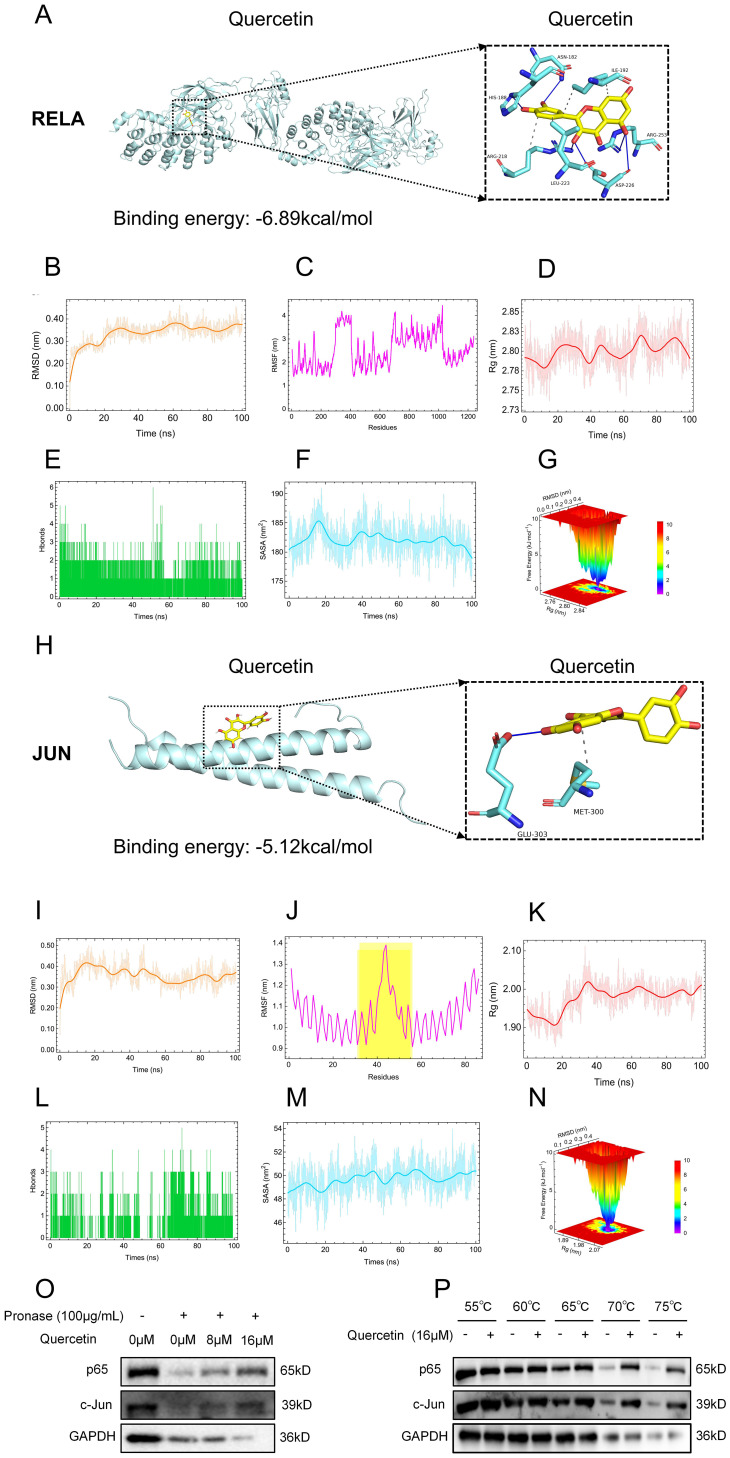
Target engagement validation of quercetin with RELA (p65) and JUN (c-Jun). **(A)** Molecular docking of quercetin with RELA (PDB: 1NFI). **(B–G)** 100 ns molecular dynamics (MD) simulation of the quercetin-RELA complex, including RMSD, RMSF, Rg, hydrogen bonds, SASA, and free energy landscape. **(H)** Molecular docking of quercetin with JUN (PDB: 5FV8). **(I–N)** 100 ns MD simulation of the quercetin-JUN complex [parameters as in **(B–G)**]. **(O)** DARTS assay demonstrating dose-dependent protection of p65 and c-Jun from pronase degradation by quercetin. **(P)** CETSA showing quercetin-enhanced thermal stability of endogenous p65 and c-Jun across a temperature gradient.

Molecular docking demonstrated a high binding affinity between quercetin and RELA (−6.89 kcal/mol) (**Figure 6A**). Molecular dynamics simulations showed that the quercetin-RELA complex stabilized with RMSD fluctuations within ~0.38 nm over 100 ns (**Figure 6B**). RMSF analysis revealed localized regions of high flexibility in RELA residues, suggesting conformational adaptability upon ligand binding while maintaining overall complex stability (**Figure 6C**). Rg stabilized at ~2.79 nm, indicating the formation of a compact structure (**Figure 6D**). Throughout the simulation, 1–6 hydrogen bonds formed between quercetin and RELA, reflecting stable specific interactions (**Figure 6E**). Although SASA fluctuated over time, stable Rg suggested consistent complex solubility (**Figure 6F**). The free energy landscape exhibited a single major energy minimum cluster, indicating a stable predominant binding conformation (**Figure 6G**). Similar robust binding dynamics were observed for the quercetin-JUN complex (−5.12 kcal/mol) (**Figures 6H–N**).

To experimentally validate this direct target engagement at the physical level, we employed DARTS and CETSA techniques. Remarkably, DARTS analysis revealed that escalating concentrations of quercetin dose-dependently protected p65 and c-Jun proteins from enzymatic degradation (**Figure 6O**; **Supplementary Figures 4A, B**). This was fully corroborated by CETSA, which demonstrated enhanced thermal stability of quercetin-bound p65 and c-Jun (**Figure 6P**; **Supplementary Figures 4C, D**). These compelling physical interaction assays confirm that quercetin directly binds to and stabilizes intracellular p65 and c-Jun (29).

### 3.3 *In Vitro* validation: suppression of pathogenic cytokines in patient-derived SSc CD4^+^ T cells

Previous studies have shown that in scleroderma, CD4^+^ T cells produce IL-17A, IFN-γ, and IL-4, which promote dermal fibroblast fibrosis, with p65 and c-Jun activation playing a key role. Our initial *in vivo* experiments suggested that the extract may inhibit p65 and c-Jun activation, thereby reducing IL-17A and IFN-γ expression in CD4^+^ T cells and alleviating skin fibrosis. To confirm this, we performed *in vitro* experiments to assess the effects on IL-17A and IFN-γ expression in CD4^+^ T cells and subsequently examined whether these modulated CD4^+^ T cells affect fibroblast fibrosis in a co-culture system.

**Figure 7:**
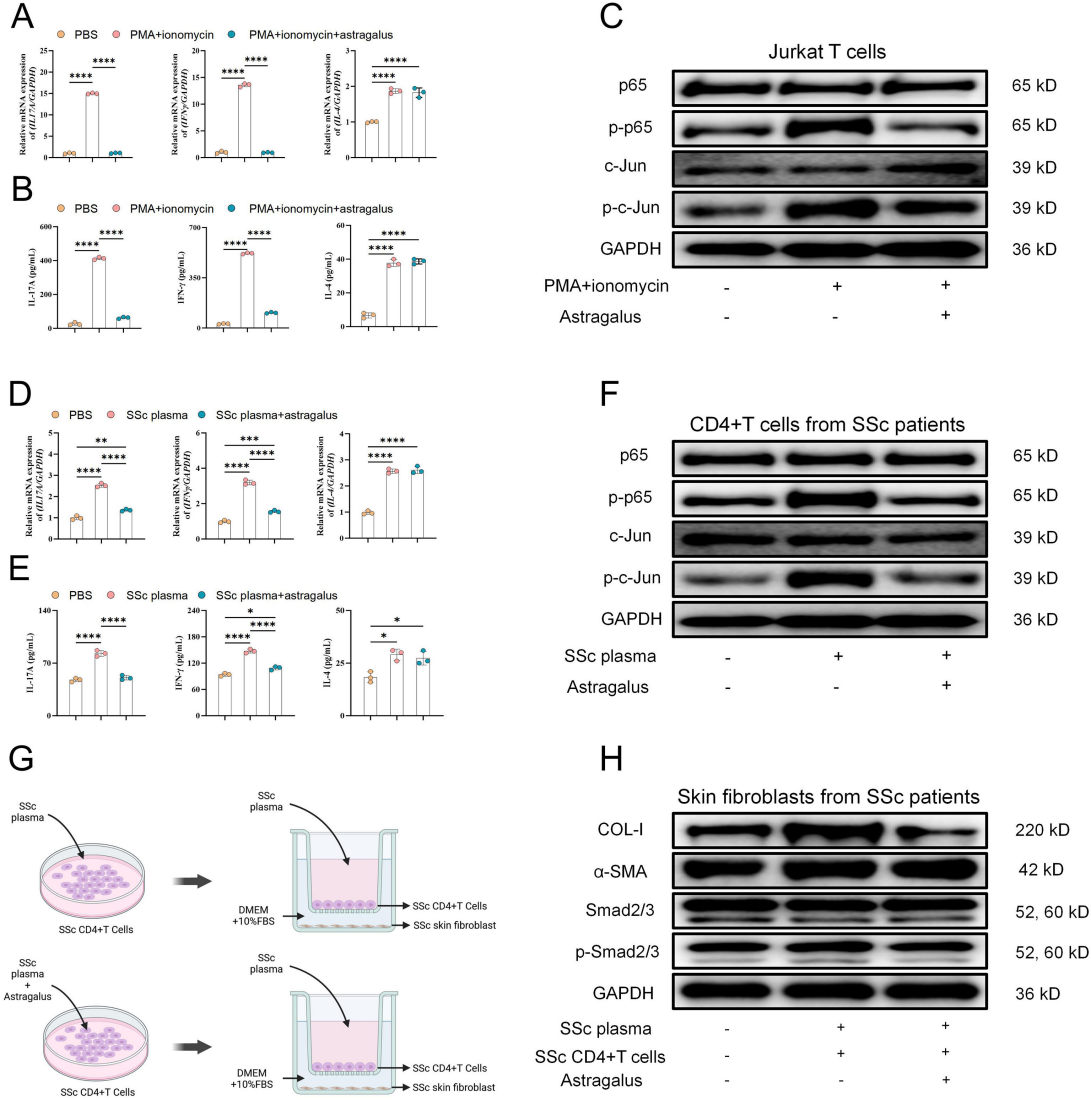
Astragalus extract inhibits IL-17/IFN-γ signaling and T cell-driven fibroblast activation. **(A–C)** qPCR, ELISA, and Western blot analysis of cytokine expression and p65/c-Jun activation in stimulated Jurkat T cells. **(D–F)** qPCR, ELISA, and Western blot evaluations of cytokines and signaling markers in primary CD4^+^ T cells from SSc patients. **(G)** Schematic of the T cell-fibroblast co-culture system. **(H)** Western blot analysis of profibrotic markers (COL-I, α-SMA, Smad2/3 and p-Smad2/3) in SSc dermal fibroblasts following co-culture. Data are mean ± SD; *p < 0.05, **p < 0.01, ***p < 0.001, ****p < 0.0001 (one-way ANOVA).

Jurkat T cells were activated with PMA and ionomycin. CCK-8 assay showed that 500 μg/mL of the extract had no cytotoxic effect; thus, this concentration was used for subsequent experiments. The treatment significantly reduced IL-17A and IFN-γ mRNA expression, as well as p65 and c-Jun phosphorylation, in activated Jurkat T cells, while IL-4 expression remained unchanged (**Figures 7A–C**; **Supplementary Figure 5A**), indicating selective inhibition of pro-inflammatory cytokines.

To validate these findings in a disease-relevant model, primary CD4^+^ T cells isolated from SSc patients were treated in the presence of SSc plasma. The treatment decreased IL-17A and IFN-γ expression at both mRNA and protein levels by approximately 50% and 40%, respectively (**Figures 7D–F**, **Supplementary Figure 5B**). Furthermore, SSc CD4^+^ T cells pretreated were co-cultured with SSc dermal fibroblasts (**Figure 7G**). This resulted in significantly reduced protein levels of fibrotic markers COL-I, α-SMA, Smad2/3, and p-Smad2/3 in fibroblasts (**Figure 7H**; **Supplementary Figure 5C**).

### 3.4 Quercetin functions as a precision immunomodulator in primary SSc CD4^+^ T cells

**Figure 8:**
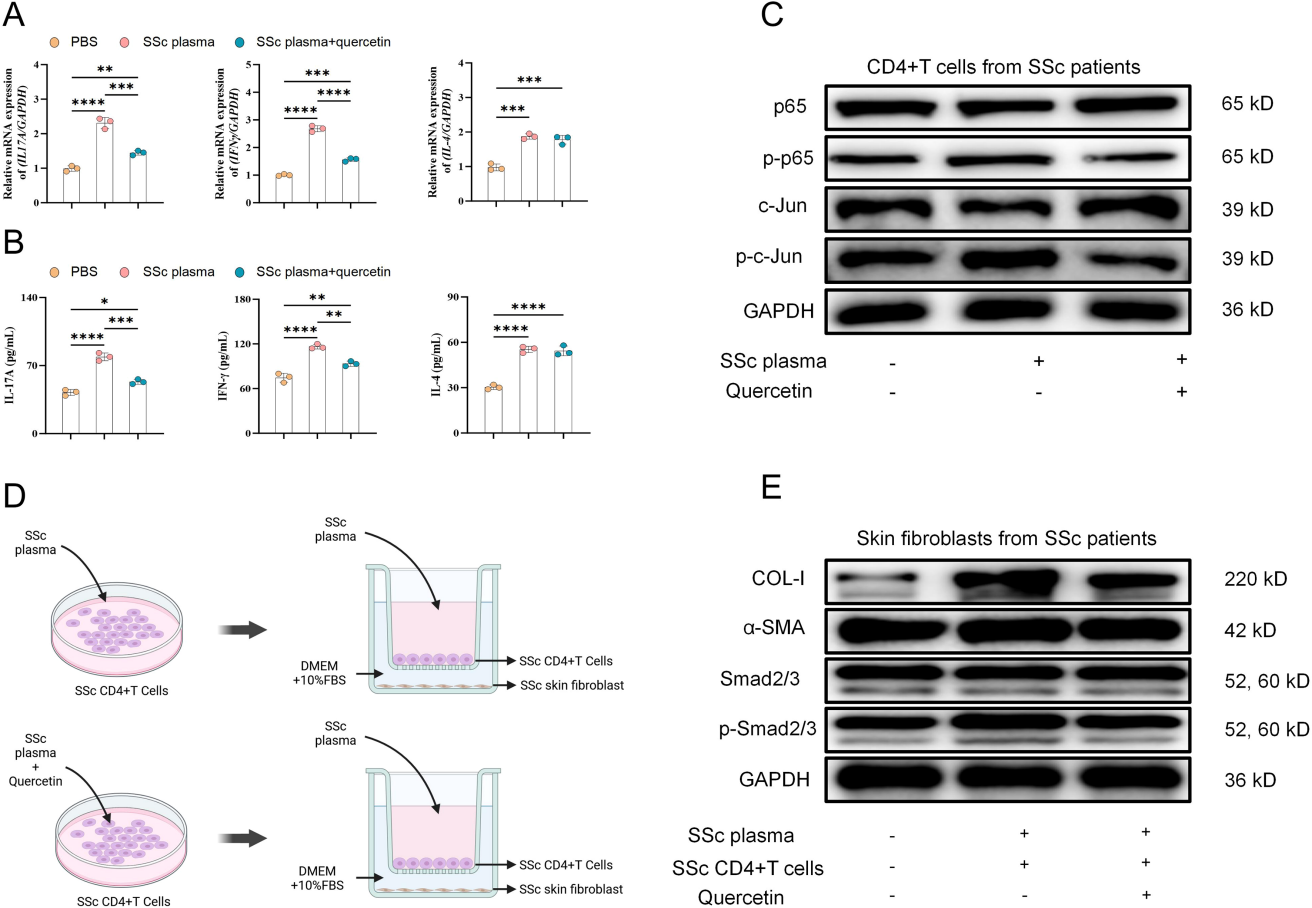
Quercetin inhibits IL-17 signaling in SSc CD4^+^ T cells and blunts fibroblast activation. **(A–C)** qPCR, ELISA, and Western blot analysis of cytokine expression and p65/c-Jun activation in SSc patient-derived CD4^+^ T cells. **(D)** Illustration of the T cell-fibroblast co-culture model. **(E)** Western blot evaluation of COL-I, α-SMA, Smad2/3 and p-Smad2/3 signaling in SSc dermal fibroblasts following co-culture with quercetin-pretreated T cells. Data are mean ± SD; *p < 0.05, **p < 0.01, ***p < 0.001, ****p < 0.0001 (one-way ANOVA).

Based on DARTS, and CETSA results, quercetin may regulate CD4^+^ T cell inflammatory responses by inhibiting p65 and c-Jun phosphorylation. To test this hypothesis in a clinical context, we treated SSc patient-derived CD4^+^ T cells with purified quercetin. Quercetin significantly suppressed IL-17A and IFN-γ expression at both mRNA and protein levels (**Figures 8A, B**) and inhibited p65 and c-Jun phosphorylation (**Figure 8C**; **Supplementary Figure 6A**).

In co-culture experiments, SSc dermal fibroblasts were cultured with quercetin-pretreated or untreated SSc CD4^+^ T cells. Fibroblasts co-cultured with quercetin-treated T cells showed significantly reduced expression of COL-I, α-SMA, Smad2/3, and p-Smad2/3 compared to controls (**Figures 8D, E**; **Supplementary Figure 6B**). These findings suggest that quercetin suppresses dermal fibroblast fibrosis by inhibiting p65 and c-Jun phosphorylation in CD4^+^ T cells, thereby reducing IL-17A and IFN-γ expression.

### 3.5 Quercetin restricts SSc progression *in vivo* by targeting the Th17-fibroblast axis

**Figure 9:**
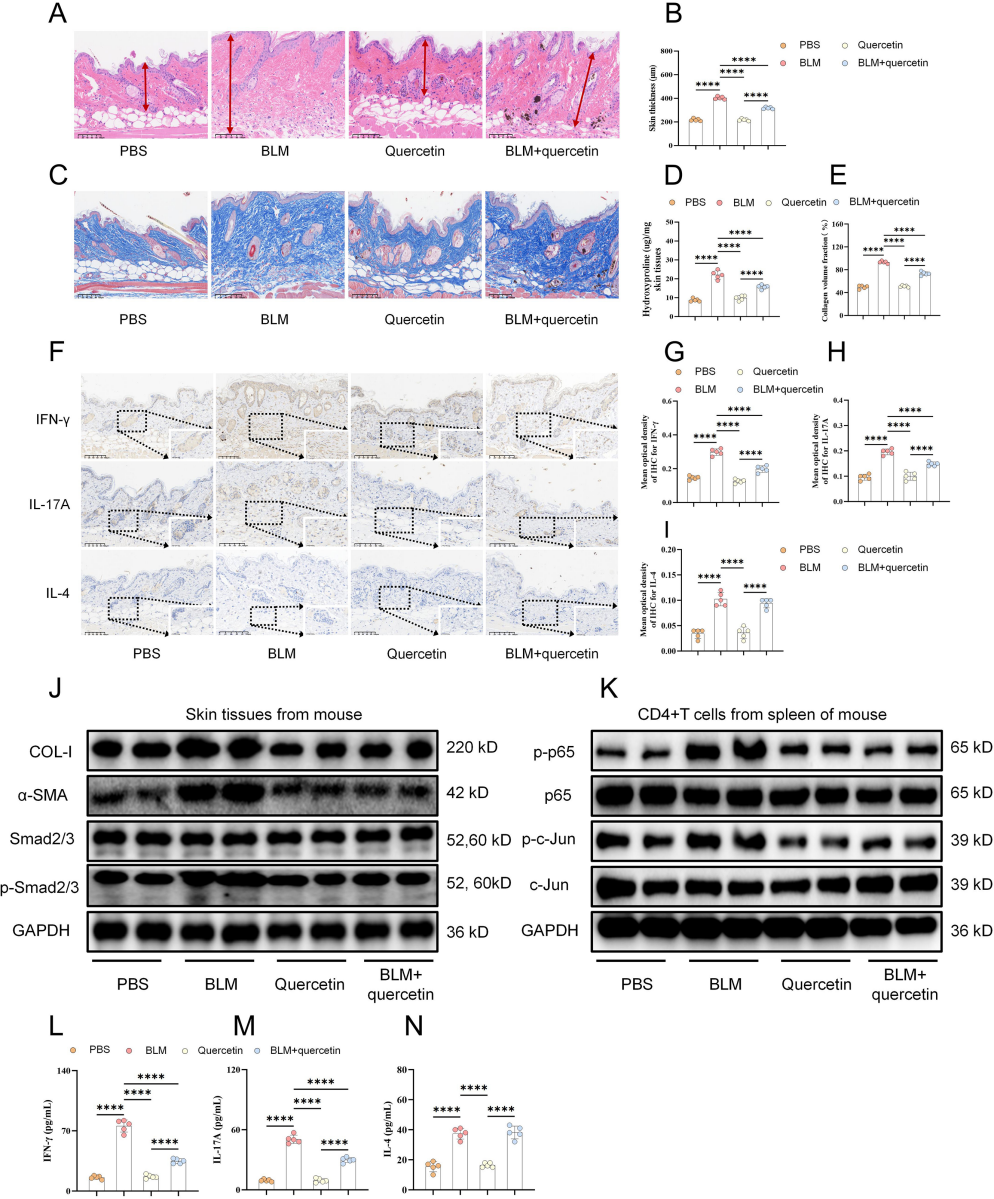
Quercetin suppresses SSc progression *in vivo* by inhibiting the IL-17 signaling axis. **(A–E)** Representative H&E and Masson’s trichrome staining of skin tissues, with quantification of dermal thickness, hydroxyproline content, and collagen volume fraction. **(F–I)** IHC analysis and quantification of IFN-γ, IL-17A, and IL-4 in skin sections. **(J, K)** Western blot analysis of profibrotic markers **(J)** and phosphorylation of p65 and c-Jun in splenic CD4^+^ T cells **(K)**. **(L–N)** ELISA quantification of plasma IFN-γ, IL-17A, and IL-4 levels. Data are mean ± SD; **p < 0.01, ***p < 0.001, ****p < 0.0001 (one-way ANOVA).

To further evaluate the therapeutic potential of the purified molecule quercetin in skin fibrosis, we administered quercetin orally to BLM-induced fibrotic mice. H&E staining showed that quercetin significantly attenuated BLM-induced skin thickening (**Figures 9A, B**). Masson’s trichrome staining revealed that quercetin markedly reduced hydroxyproline and collagen content in the skin (**Figures 9C–E**). Immunohistochemical analysis demonstrated that quercetin suppressed BLM-induced IL-17A and IFN-γ protein expression in skin tissues (**Figures 9F–I**). Western blot analysis indicated that quercetin downregulated COL-I, α-SMA, Smad2/3, and p-Smad2/3 in the skin of BLM-treated mice (**Figure 9J**; **Supplementary Figure 7A**). Additionally, quercetin inhibited BLM-induced phosphorylation of p65 and c-Jun in splenic CD4^+^ T cells (**Figure 9K**; **Supplementary Figure 7B**). ELISA of plasma samples showed that quercetin reduced IL-17A and IFN-γ levels, while IL-4 remained unchanged (**Figures 9L–N**).

## 4 Discussion

Systemic sclerosis (SSc) is a devastating autoimmune pathology in which immune dysregulation and unremitting fibroblast activation are major contributors to mortality. Accumulating evidence indicates that aberrant CD4^+^ T cell function plays a central role in SSc pathogenesis, both initiating and perpetuating inflammation and fibrosis (26–28). Among CD4^+^ T cell subsets, Th17 cells have garnered particular attention. Th17 cells drive inflammatory responses by secreting IL-17A and IFN-γ, promoting fibroblast activation and excessive extracellular matrix deposition (30). Extensive research has established the IL-17 signaling pathway as a key mediator in SSc, with elevated IL-17A levels closely correlated with disease severity (31–33). These findings have spurred immense interest in targeting immune pathways for SSc treatment. Notably, the anti-IL-17A receptor monoclonal antibody brodalumab has been investigated in clinical trials, rapidly and persistently reducing the modified Rodnan skin score (mRSS) (34). These studies underscore the immense therapeutic potential of precision interventions targeting the IL-17A cascade in SSc.

Historically, botanical extracts such as Astragalus have exhibited well-documented immunomodulatory and anti-fibrotic properties across multiple organ models (35–38). For instance, certain active fractions have been shown to restore Treg/Th17 balance by inhibiting the p65 signaling pathway, thereby regulating IL-17-mediated immune responses (39). However, the translation of these broad botanical phenomena into targeted SSc therapies requires the precise identification of specific bioactive small molecules and their intracellular targets within pathogenic immune cells.

In this study, we employed an integrated approach to deconstruct this botanical efficacy, successfully pinpointing the IL-17 signaling cascade as the central therapeutic axis. This was unequivocally validated in our bleomycin-induced murine model, where the treatment remarkably reversed dermal fibrosis and structural matrix deposition. Crucially, our *in vitro* investigations utilizing highly clinically relevant CD4^+^ T cells derived directly from SSc patients confirmed that this intervention potently restrained pathogenic Th17 differentiation and blunted the transcription of IL-17A and IFN-γ, subsequently abrogating the fibrotic activation of co-cultured dermal fibroblasts.

Advancing beyond whole-extract phenomena, our rigorous target-engagement assays (DARTS and CETSA) successfully identified quercetin as the pivotal bioactive mediator. Quercetin directly physically engages and stabilizes RELA and c-Jun, key transcriptional effectors of the IL-17 pathway. Given the established roles of NF-κB and JUN signaling in promoting pro-inflammatory gene expression (40, 41), these findings indicate that quercetin effectively short-circuits the pro-inflammatory transcription program. Our findings align with emerging evidence highlighting quercetin as a broad-spectrum precision modulator of pathogenic T-cell subsets. For instance, recent studies have demonstrated that quercetin can induce cellular senescence in follicular helper T (Tfh) cells and suppress the production of inflammatory cytokines, thereby alleviating disease progression in systemic lupus erythematosus (18). Furthermore, quercetin has been shown to inhibit the activation and differentiation of Th2 cells, effectively mitigating airway hyperresponsiveness and allergic inflammation (19). Consistent with these diverse immunomodulatory properties, while previous literature has documented quercetin’s general anti-fibrotic effects (42–47) and its ability to suppress IL-17A in other contexts (48–54), our study is the first to conclusively establish that quercetin ameliorates SSc-specific skin fibrosis primarily by dismantling the IL-17 signaling axis directly within CD4^+^ T cells.

The transcription factors p65 (RELA) and c-Jun serve as critical intracellular hubs regulating inflammatory responses. Mice harboring alterations in c-Jun or p65 demonstrate significant immunological defects (55, 56), highlighting their indispensability in immune homeostasis. In the context of SSc, the hyperactivation of these factors contributes critically to skin inflammation and fibrotic remodeling (57, 58). Our data reveal that quercetin-mediated physical stabilization and subsequent functional inhibition of p65 and c-Jun effectively dampens the IL-17 inflammatory cascade, thereby alleviating chronic skin inflammation and disrupting the pathogenic T cell-fibroblast crosstalk. Previous studies have found that quercetin can inhibit the expression levels of inflammatory cytokines such as IL-4 and IL-6 in Graft-versus-host disease (GVHD) mice, though the underlying mechanisms remain elusive (21). Consequently, the present study represents the first systematic evaluation of quercetin’s effects on skin inflammation and fibrosis in SSc, as well as its inhibitory impact on the IL-17 signaling pathway. These findings suggest that quercetin possesses significant potential as a therapeutic agent for SSc. However, as a lipophilic compound, quercetin’s low solubility presents a challenge for its pharmacological development (23). Notably, several studies have utilized liposomal encapsulation for natural products like quercetin to enhance their hydrophilicity and stability, thereby achieving superior therapeutic outcomes (59). In future research, we aim to employ these liposome-encapsulated quercetin formulations in both cell and mouse models to further investigate their efficacy in mitigating inflammation and fibrosis across multiple tissues and organs in SSc.

Furthermore, γδ T cells represent a crucial cell population in the pathogenesis of SSc. Existing evidence suggests that γδ T cells from SSc patients exhibit enhanced cytotoxicity, which contributes significantly to endothelial cell injury (60). Among them, γδ T17 cells-characterized by their specific secretion of the pro-inflammatory cytokine IL-17-are potent drivers of skin inflammation and are established as key factors in the development of psoriasis (61). Given that IL-17A plays a pivotal role in promoting skin fibrosis in SSc, γδ T17 cells likely exert a substantial influence on the fibrotic process (31–34). In subsequent studies, we intend to investigate whether the frequency and functional activity of γδ T17 cells are elevated in SSc patients. We also aim to elucidate their specific effects on endothelial cells and fibroblasts, while evaluating whether quercetin can effectively suppress the activation and pathogenic functions of these γδ T17 cells.

We acknowledge certain limitations in this study. While DARTS and CETSA provide robust evidence of direct target engagement, the precise sub-molecular architecture of the quercetin-RELA/c-Jun binding interfaces warrants high-resolution elucidation, potentially via X-ray crystallography or cryo-electron microscopy. Furthermore, owing to the embryonic lethality associated with systemic c-Jun or p65 ablation, comprehensive *in vivo* genetic rescue models could not be deployed. Future studies utilizing T cell-specific conditional knockout (cKO) murine models will be instrumental in further dissecting these intracellular mechanisms.

## 5 Conclusions

In conclusion, this study provides compelling evidence that quercetin functions as a precision small-molecule immunomodulator, orchestrating profound anti-inflammatory and anti-fibrotic effects in SSc. By directly and physically engaging the p65 (RELA) and c-Jun transcriptional complex, quercetin suppresses the pathogenic IL-17 signaling cascade and uncouples aberrant T-cell activation from downstream fibrotic responses. Collectively, these findings highlight the RELA/c-Jun interface as a critical therapeutic vulnerability in SSc, establishing quercetin as a highly promising candidate for the targeted immunotherapy of immune-driven fibrotic diseases.

## Data Availability

The original contributions presented in the study are included in the article/**Supplementary Material**, further inquiries can be directed to the corresponding author/s.

## References

[1] Denton CP Khanna D . Systemic sclerosis. Lancet. (2017) 390:1685–99. doi: 10.1383/medc.30.10.36.28247 28413064

[2] Bergamasco A Hartmann N Wallace L Verpillat P . Epidemiology of systemic sclerosis and systemic sclerosis-associated interstitial lung disease. Clin Epidemiol. (2019) 11:257–73. doi: 10.2147/CLEP.S191418 PMC6497473 31114386

[3] Jerjen R Nikpour M Krieg T Denton CP Saracino AM . Systemic sclerosis in adults. Part I: Clinical features and pathogenesis. J Am Acad Dermatol. (2022) 87:937–54. doi: 10.1016/j.jaad.2021.10.065. PMID: 35131402

[4] Wang C Oishi K Kobayashi T Fujii K Horii M Fushida N . The role of TLR7 and TLR9 in the pathogenesis of systemic sclerosis. Int J Mol Sci. (2024) 25:6133. doi: 10.3390/ijms25116133. PMID: 38892317 PMC11172923

[5] Balanescu P Ladaru A Balanescu E Nicolau A Baicus C Dan GA . IL-17, IL-6 and IFN-γ in systemic sclerosis patients. Rom J Intern Med. (2015) 53:44–9. doi: 10.1515/rjim-2015-0006 26076560

[6] Seki N Tsujimoto H Tanemura S Ishigaki S Takei H Sugahara K . Th17/IL-17A axis is critical for pulmonary arterial hypertension (PAH) in systemic sclerosis (SSc): SSc patients with high levels of serum IL-17A exhibit reduced lung functions and increased prevalence of PAH. Cytokine. (2014) 176:156534. doi: 10.1016/j.cyto.2024.156534. PMID: 38354516

[7] Chong WP Mattapallil MJ Raychaudhuri K Bing SJ Wu S Zhong Y . The cytokine IL-17A limits Th17 pathogenicity via a negative feedback loop driven by autocrine induction of IL-24. Immunity. (2020) 53:384–397.e5. doi: 10.1016/j.immuni.2020.06.022. PMID: 32673565 PMC7362799

[8] Lei L Zhao C Qin F He ZY Wang X Zhong XN . Th17 cells and IL-17 promote the skin and lung inflammation and fibrosis process in a bleomycin-induced murine model of systemic sclerosis. Clin Exp Rheumatol. (2016) 34:14–22. 26750756

[9] Mirsaeidi M Barletta P Glassberg MK . Interstitial lung disease associated with systemic sclerosis (SSc-ILD). Respir Res. (2019) 20:13. doi: 10.1186/s12931-019-0980-7 30658650 PMC6339436

[10] Perelas A Silver RM Arrossi AV Highland KB . Systemic sclerosis-associated interstitial lung disease. Lancet Respir Med. (2020) 8:304–20. doi: 10.1016/s2213-2600(19)30480-1. PMID: 32113575

[11] Lescoat A Roofeh D Kuwana M Lafyatis R Allanore Y Khanna D . Therapeutic approaches to systemic sclerosis: Recent approvals and future candidate therapies. Clin Rev Allergy Immunol. (2023) 64:239–61. doi: 10.1007/s12016-021-08891-0. PMID: 34468946 PMC9034469

[12] Bukiri H Volkmann ER . Current advances in the treatment of systemic sclerosis. Curr Opin Pharmacol. (2022) 64:102211. doi: 10.1016/j.coph.2022.102211. PMID: 35447517 PMC9466985

[13] Bayat M Nahand JS . CAR-engineered cell therapies: Current understandings and future perspectives. Mol BioMed. (2026) 7:7. doi: 10.1186/s43556-025-00401-4. PMID: 41559435 PMC12819963

[14] Bale S Pulivendala G Godugu C . Withaferin A attenuates bleomycin-induced scleroderma by targeting FoxO3a and NF-κβ signaling: Connecting fibrosis and inflammation. Biofactors. (2018) 44:507–17. doi: 10.1002/biof.1446. PMID: 30367690

[15] Assar S Khazaei H Naseri M El-Senduny F Momtaz S Farzaei MH . Natural formulations: Novel viewpoint for scleroderma adjunct treatment. J Immunol Res. (2021) 2021:9920416. doi: 10.1155/2021/9920416. PMID: 34258301 PMC8253639

[16] Abd Elkader HT Essawy AE Al-Shami AS . Astragalus mongholicus: A review of its anti-fibrosis properties. Front Pharmacol. (2022) 13:976561. doi: 10.3389/fphar.2022.976561 36160396 PMC9490009

[17] Wei Y Qi M Liu C Li L . Astragalus polysaccharide attenuates bleomycin-induced pulmonary fibrosis by inhibiting TLR4/ NF-κB signaling pathway and regulating gut microbiota. Eur J Pharmacol. (2023) 944:175594. doi: 10.1016/j.ejphar.2023.175594. PMID: 36804541

[18] Xiong F Shen K Long D Zhou S Ruan P Xin Y . Quercetin ameliorates lupus symptoms by promoting the apoptosis of senescent Tfh cells via the Bcl-2 pathway. Immun Ageing. (2024) 21:69. doi: 10.1186/s12979-024-00474-9. PMID: 39407236 PMC11476537

[19] Dong Y Liu L Zhang X Zheng H Liu Y Zhang A . Quercetin improves macrophage immune regulatory functions to alleviate airway Th2 polarization. Immunol Lett. (2025) 275:107030. doi: 10.1016/j.imlet.2025.107030. PMID: 40316181

[20] Qi Q Mao Y Yi J Li D Zhu K Cha X . Anti-fibrotic effects of astragaloside IV in systemic sclerosis. Cell Physiol Biochem. (2014) 34:2105–16. doi: 10.1159/000366405. PMID: 25562158

[21] Raman D Chêne C Nicco C Jeljeli M Eu JQ Clément MV . Therapeutic potential of a senolytic approach in a murine model of chronic GVHD. Biol (Basel). (2023) 12:647. doi: 10.3390/biology12050647. PMID: 37237461 PMC10215844

[22] Deng CC Xu XY Zhang Y Liu LC Wang X Chen JY . Single-cell RNA-seq reveals immune cell heterogeneity and increased Th17 cells in human fibrotic skin diseases. Front Immunol. (2025) 15:1522076. doi: 10.3389/fimmu.2024.1522076. PMID: 39872534 PMC11769821

[23] Liu Q Yang X Sun J Yu F Zhang H Gao J . Zheng. Size-dependent biological effects of quercetin nanocrystals. Molecules. (2019) 24:1438. doi: 10.3390/molecules24071438. PMID: 30979064 PMC6479833

[24] Catauro M Bollino F Papale F Piccolella S Pacifico S . Sol-gel synthesis and characterization of SiO2/PCL hybrid materials containing quercetin as new materials for antioxidant implants. Mater Sci Eng C Mater Biol Appl. (2016) 58:945–52. doi: 10.1016/j.msec.2015.09.054. PMID: 26478390

[25] Košinová P Berka K Wykes M Otyepka M Trouillas P . Positioning of antioxidant quercetin and its metabolites in lipid bilayer membranes: Implication for their lipid-peroxidation inhibition. J Phys Chem B. (2012) 116:1309–18. doi: 10.1021/jp208731g 22201287

[26] Jin W Zheng Y Zhu P . T cell abnormalities in systemic sclerosis. Autoimmun Rev. (2022) 21:103185. doi: 10.1016/j.autrev.2022.103185. PMID: 36031049

[27] Liu M Wu W Sun X Yang J Xu J Fu W . New insights into CD4(+) T cell abnormalities in systemic sclerosis. Cytokine Growth Factor Rev. (2016) 28:31–6. doi: 10.1016/j.cytogfr.2015.12.002. PMID: 26724976

[28] Maehara T Kaneko N Perugino CA Mattoo H Kers J Allard-Chamard H . Cytotoxic CD4+ T lymphocytes may induce endothelial cell apoptosis in systemic sclerosis. J Clin Invest. (2020) 130:2451–64. doi: 10.1172/jci131700. PMID: 31990684 PMC7190971

[29] Wu Z Wang Y Wang R Gao R Li S Tu B . Paeoniflorin alleviates hypobaric hypoxia-triggered lung injury through targeting MEK2 to modulate ERK2-SGK1 signaling. Acta Pharm Sin B. (2026) 16:1466–88. doi: 10.1016/j.apsb.2025.12.024. PMID: 41909739 PMC13031058

[30] Van Caam A Vonk M van Den Hoogen F van Lent P van Der Kraan P . Unraveling SSc pathophysiology; The myofibroblast. Front Immunol. (2018) 13:2452. doi: 10.3389/fimmu.2018.02452. PMID: 30483246 PMC6242950

[31] Gabsi A Heim X Dlala A Gati A Sakhri H Abidi A . TH17 cells expressing CD146 are significantly increased in patients with systemic sclerosis. Sci Rep. (2019) 9:17721. doi: 10.1038/s41598-019-54132-y. PMID: 31776424 PMC6881361

[32] Almanzar G Klein M Schmalzing M Hilligardt D El Hajj N Kneitz H . Disease manifestation and inflammatory activity as modulators of Th17/Treg balance and RORC/FoxP3 methylation in systemic sclerosis. Int Arch Allergy Immunol. (2016) 171:141–54. doi: 10.1159/000450949. PMID: 27902985

[33] Balanescu P Balanescu E Balanescu A . IL-17 and Th17 cells in systemic sclerosis: A comprehensive review. Rom J Intern Med. (2017) 55:198–204. doi: 10.1515/rjim-2017-0027 28704201

[34] Fukasawa T Yoshizaki A Ebata S Fukayama M Kuzumi A Norimatsu Y . Interleukin-17 pathway inhibition with brodalumab in early systemic sclerosis: Analysis of a single-arm, open-label, phase 1 trial. J Am Acad Dermatol. (2023) 89:366–9. doi: 10.1016/j.jaad.2023.02.061. PMID: 36997069

[35] Lan J Nie W Bi Z Zeng R Li Z Zhang T . Astragalus polysaccharide-based nano-platforms loading PTX to boost chemo-immunotherapy for triple-negative breast cancer with intrinsic GLUT-targeting ability and immunoregulatory activity. J Nanobiotechnol. (2025) 23:628. doi: 10.1186/s12951-025-03708-0. PMID: 41053818 PMC12498460

[36] Jiang H Zhou R An L Guo J Hou X Tang J . Exploring the role and mechanism of Astragalus membranaceus and radix paeoniae rubra in idiopathic pulmonary fibrosis through network pharmacology and experimental validation. Sci Rep. (2023) 13:10110. doi: 10.1038/s41598-023-36944-1. PMID: 37666859 PMC10477296

[37] Zhang X Qu H Yang T Liu Q Zhou H . Astragaloside IV attenuate MI-induced myocardial fibrosis and cardiac remodeling by inhibiting ROS/caspase-1/GSDMD signaling pathway. Cell Cycle. (2022) 21:2309–22. doi: 10.1080/15384101.2022.2093598. PMID: 35770948 PMC9586672

[38] Dan L Hao Y Song H Wang T Li J He X . Efficacy and potential mechanisms of the main active ingredients of astragalus mongholicus in animal models of liver fibrosis: A systematic review and meta-analysis. J Ethnopharmacol. (2024) 319:117198. doi: 10.1016/j.jep.2023.117198. PMID: 37722514

[39] He X Liu L Luo X Zhu J Yang H Wang J . Astragalus polysaccharide relieves inflammatory responses in Guinea pigs with allergic rhinitis via ameliorating NF-kB-mediated Treg/Th17 imbalance. Am J Rhinol Allergy. (2022) 36:638–48. doi: 10.1177/19458924221098847. PMID: 35585694

[40] Zhou TY Guo YY Jing QQ Wei MY Xu WF Gu YC . Semisynthesis and biological evaluation of 17-hydroxybrevianamide N derivatives as anti-inflammatory agents by mediating NF-κB and MAPK signaling pathways. Eur J Med Chem. (2025) 290:117541. doi: 10.1016/j.ejmech.2025.117541. PMID: 40174263

[41] Huangfu L Li R Huang Y Wang S . The IL-17 family in diseases: From bench to bedside. Signal Transduct Target Ther. (2023) 8:402. doi: 10.1038/s41392-023-01620-3. PMID: 37816755 PMC10564932

[42] Horton JA Li F Chung EJ Hudak K White A Krausz K . Quercetin inhibits radiation-induced skin fibrosis. Radiat Res. (2013) 180:205–15. doi: 10.1667/rr3237.1. PMID: 23819596 PMC4281888

[43] Cho JW Cho SY Lee SR Lee KS . Onion extract and quercetin induce matrix metalloproteinase-1 *in vitro* and *in vivo*. Int J Mol Med. (2010) 25:347–52. 20127038

[44] Xia L Cai J Zeng X Shu Q Hu Q Rao S . miR-423-5p/NDUFS7-mediated mitochondrial function modulation contributes to quercetin-induced attenuation of pulmonary fibrosis via extracellular matrix remodeling regulation. Non-coding RNA Res. (2026) 18:65–76. doi: 10.1016/j.ncrna.2026.01.009. PMID: 41631271 PMC12860696

[45] Yong W Li Z Xu W Gao D Sha B Jin Y . Quercetin ameliorates renal injury by promoting UCP1-mediated alleviation of lipid accumulation in diabetic kidney disease. Phytomedicine. (2025) 147:157213. doi: 10.1016/j.phymed.2025.157213. PMID: 40907404

[46] Lin DW Jiang YW Wu C Zhang H Li YZ Wang YS . Quercetin alleviates cardiac fibrosis via regulating the SIRT3 signaling pathway. Cardiovasc Drugs Ther. (2025) 39:737–48. doi: 10.1007/s10557-024-07658-x. PMID: 39680328

[47] Lan Y Dong C Wu M Yuan R Yang K Yang Z . Quercetin ameliorates epithelial-mesenchymal transition and inflammation by targeting FSTL1 and modulating the NF-kappaB pathway in pulmonary fibrosis. Front Pharmacol. (2025) 16:1594757. doi: 10.3389/fphar.2025.1594757. PMID: 40808692 PMC12343526

[48] An Y Zhao R Liu W Wei C Jin L Zhang M . Quercetin through miR-147-5p/Clip3 axis reducing Th17 cell differentiation to alleviate periodontitis. Regener Ther. (2024) 27:496–505. doi: 10.1016/j.reth.2024.04.016 PMC11096707 38756701

[49] Wang J Wang Z Zhao Y Bai L Wei Y Huang T . Molecular mechanism of quercetin in treating RA-ILD based on network pharmacology, molecular docking, and experimental validation. Naunyn Schmiedebergs Arch Pharmacol. (2024) 397:3077–92. doi: 10.1007/s00210-023-02772-3. PMID: 37878048

[50] Chen Y Zhang M Li W Wang X Chen X Wu Y . Drug repurposing based on the similarity gene expression signatures to explore for potential indications of quercetin: A case study of multiple sclerosis. Front Chem. (2023) 11:1250043. doi: 10.3389/fchem.2023.1250043. PMID: 37744058 PMC10514366

[51] Jiang D Xiao H Zheng X Dong R Zhang H Dai H . Interleukin-17A plays a key role in pulmonary fibrosis following Propionibacterium acnes-induced sarcoidosis-like inflammation. Exp Biol Med. (2023) 248:1181–90. doi: 10.1177/15353702231182224 PMC10621476 37452708

[52] Bitterman D Wang JY Collins A Zafar K Kabakova M Patel P . The role of IL-17 and Th17 cells in keloid pathogenesis. Arch Dermatol Res. (2024) 316:626. doi: 10.1007/s00403-024-03352-y. PMID: 39276195

[53] Dabbaghizadeh A Dion J Maali Y Fouda A Bédard N Evaristo G . Novel RORgammat inverse agonists limit IL-17-mediated liver inflammation and fibrosis. J Immunol. (2025) 214:1321–31. doi: 10.1093/jimmun/vkaf014. PMID: 40073158 PMC12207071

[54] Huang L . The role of IL-17 family cytokines in cardiac fibrosis. Front Cardiovasc Med. (2024) 11:1470362. doi: 10.3389/fcvm.2024.1470362. PMID: 39502194 PMC11534612

[55] Thépot D Weitzman JB Barra J Segretain D Stinnakre MG Babinet C . Targeted disruption of the murine junD gene results in multiple defects in male reproductive function. Development. (2000) 127:143–53. doi: 10.1242/dev.127.1.143 10654608

[56] Passegue E Wagner EF Weissman IL . JunB deficiency leads to a myeloproliferative disorder arising from hematopoietic stem cells. Cell. (2004) 119:431–43. doi: 10.1016/j.cell.2004.10.010 15507213

[57] Wang Y Wang L Wen X Hao D Zhang N He G . NF-kappaB signaling in skin aging. Mech Ageing Dev. (2019) 184:111160. doi: 10.1016/j.mad.2019.111160. PMID: 31634486

[58] Reck M Baird DP Veizades S Sutherland C Bell RMB Hur H . Multiomic analysis of human kidney disease identifies a tractable inflammatory and pro-fibrotic tubular cell phenotype. Nat Commun. (2025) 16:4745. doi: 10.1038/s41467-025-59997-4. PMID: 40399382 PMC12095627

[59] Chen J Chen J Yu P Yang C Xia C Deng J . A novel quercetin encapsulated glucose modified liposome and its brain-target antioxidative neuroprotection effects. Molecules. (2024) 29:607. doi: 10.3390/molecules29030607. PMID: 38338352 PMC10856503

[60] Kahaleh MB Fan PS Otsuka T . Gammadelta receptor bearing T cells in scleroderma: enhanced interaction with vascular endothelial cells *in vitro*. Clin Immunol. (1999) 91:188–95. doi: 10.1006/clim.1999.4694. PMID: 10227811

[61] Kim SH Oh J Roh WS Park J Chung KB Lee GH . Pellino-1 promotes intrinsic activation of skin-resident IL-17A-producing T cells in psoriasis. J Allergy Clin Immunol. (2023) 151:1317–28. doi: 10.26226/m.641999d4b9b42e0013e5917c 36646143

