# Supplementary material for: Quercetin attenuates skin inflammation and fibrosis in systemic sclerosis by targeting the RELA/c-Jun axis to suppress th17 cell responses
Source: Front Immunol. 2026 Jun 3;17:1863530. doi: 10.3389/fimmu.2026.1863530 (PMC13272162; doi:10.3389/fimmu.2026.1863530)
Supplement: Supplementary file 1 [file Table1.docx]

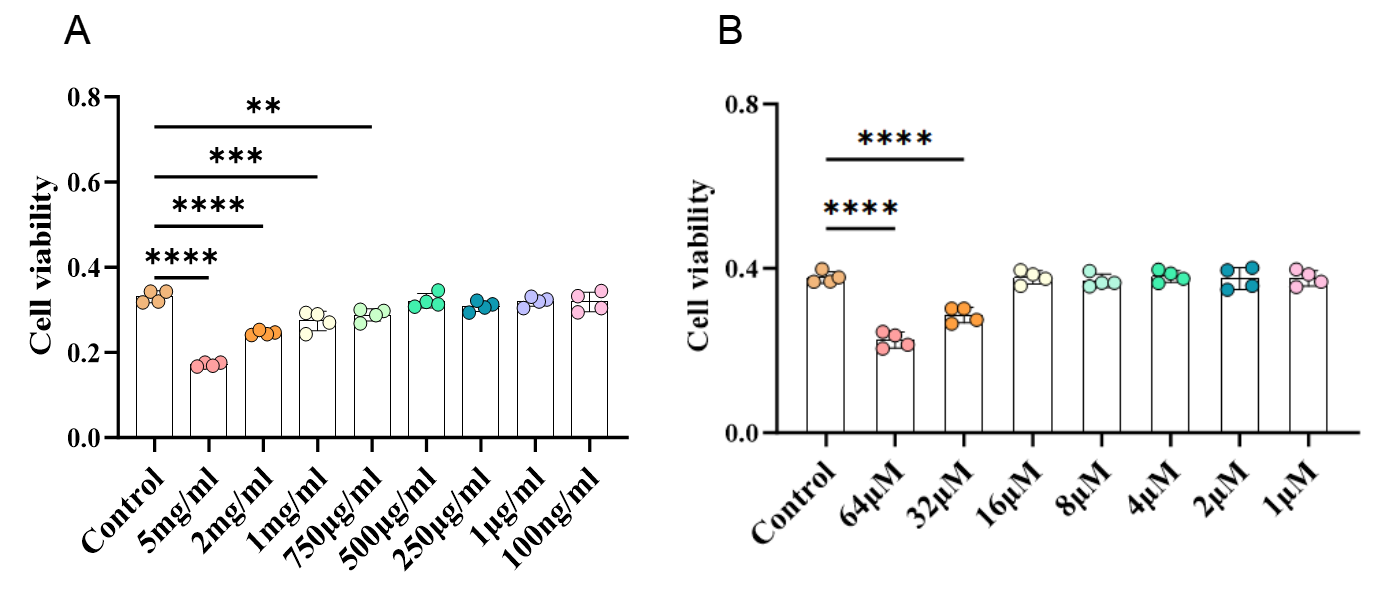


**Supplementary Figure 1. Effects of Astragalus extract and quercetin on T cell viability.** (A–B) CCK-8 assay of T cell proliferation following treatment with varying concentrations of Astragalus extract (A) and quercetin (B). Data are mean ± SD; p < 0.01, *** p < 0.001, **** p < 0.0001 (one-way ANOVA).

**
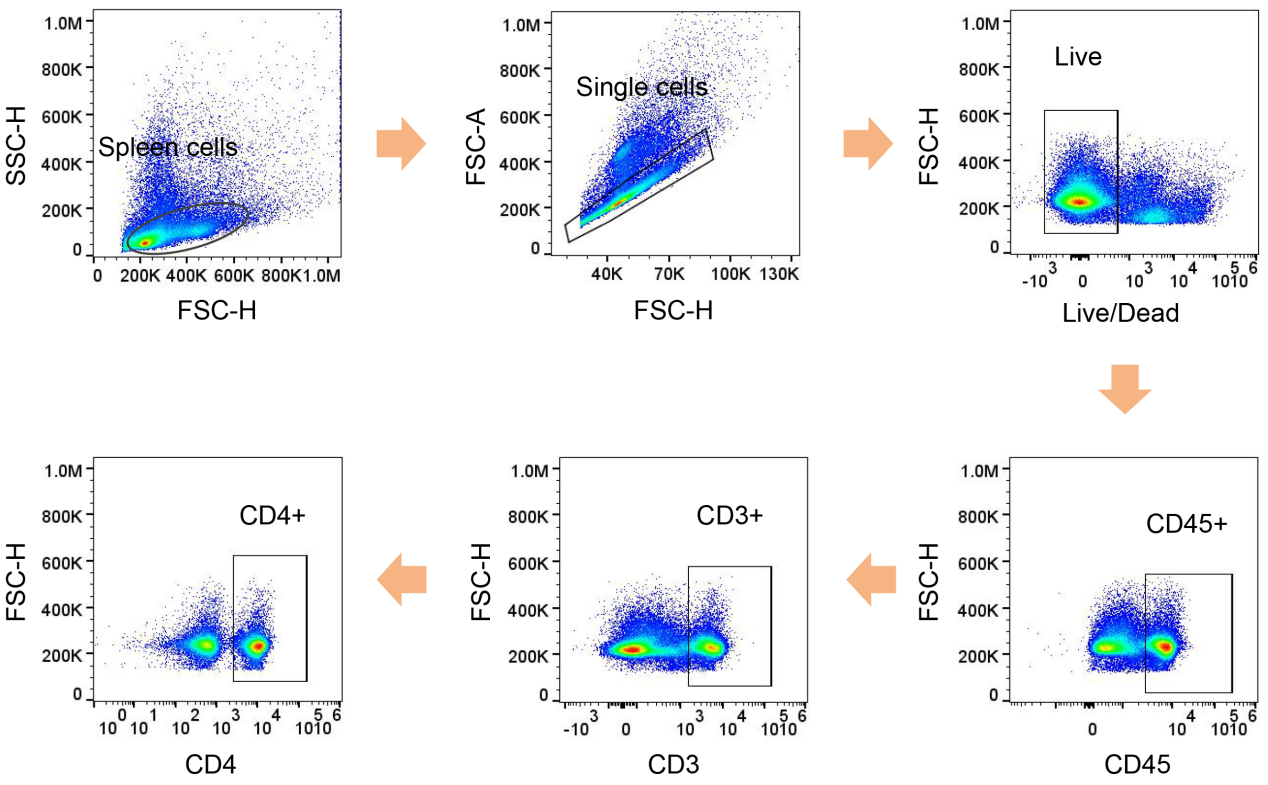
**

**Supplementary figure 2. Flow Cytometry Gating Strategy for Mouse Spleen CD4+ T Cells.**


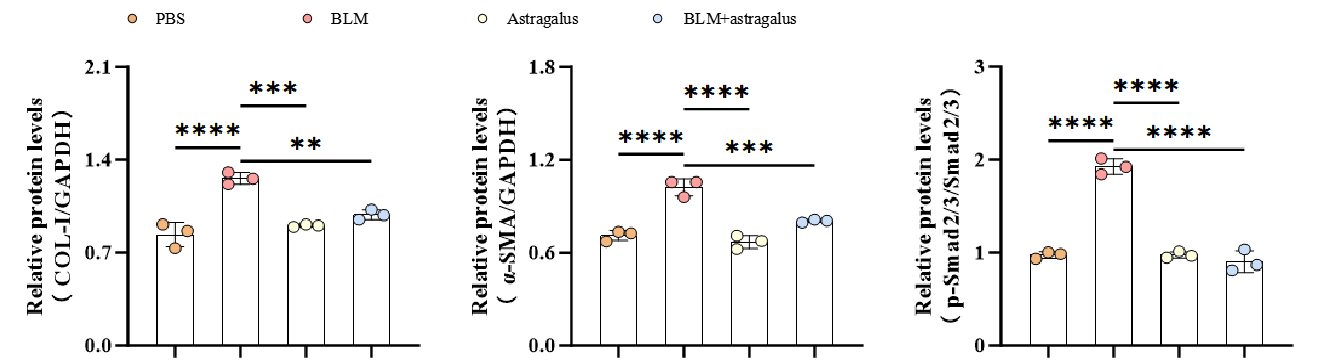


**Supplementary Figure 3. Densitometric quantification of Western blot results from Figure 3F.** (A–C) Quantitative analysis of COL-I (A), α-SMA (B), and p-Smad2/3 (C) protein expression levels. Data are mean ± SD; p < 0.01, *** p < 0.001, **** p < 0.0001 (one-way ANOVA).


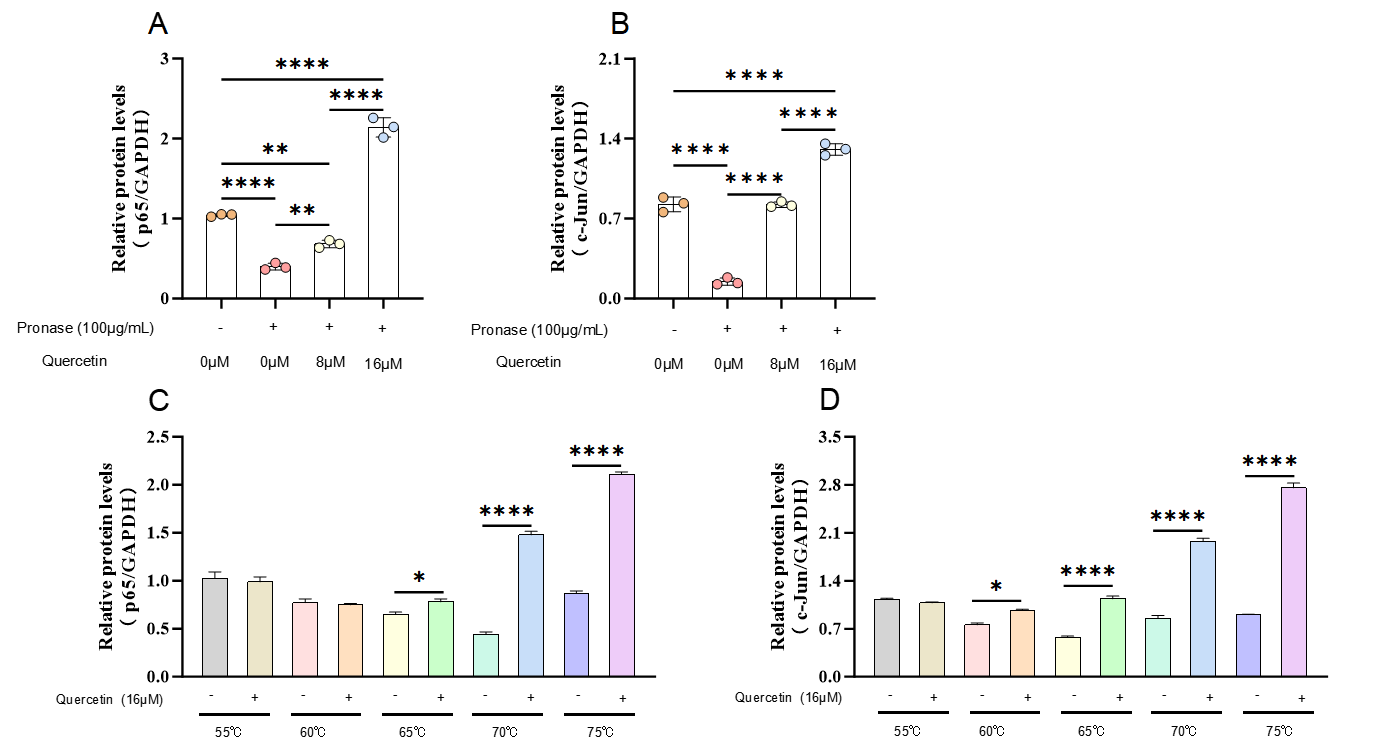


**Supplementary Figure 4. Densitometric quantification of DARTS and CETSA results from Figures 6O and 6P.** (A–B) Quantification of p65 (A) and c-Jun (B) protein levels from the DARTS assay in Figure 6O. (C–D) Quantification of p65 (C) and c-Jun (D) protein levels from the CETSA in Figure 6P. Data are mean ± SD; p < 0.01, *** p < 0.001, **** p < 0.0001 (one-way ANOVA).


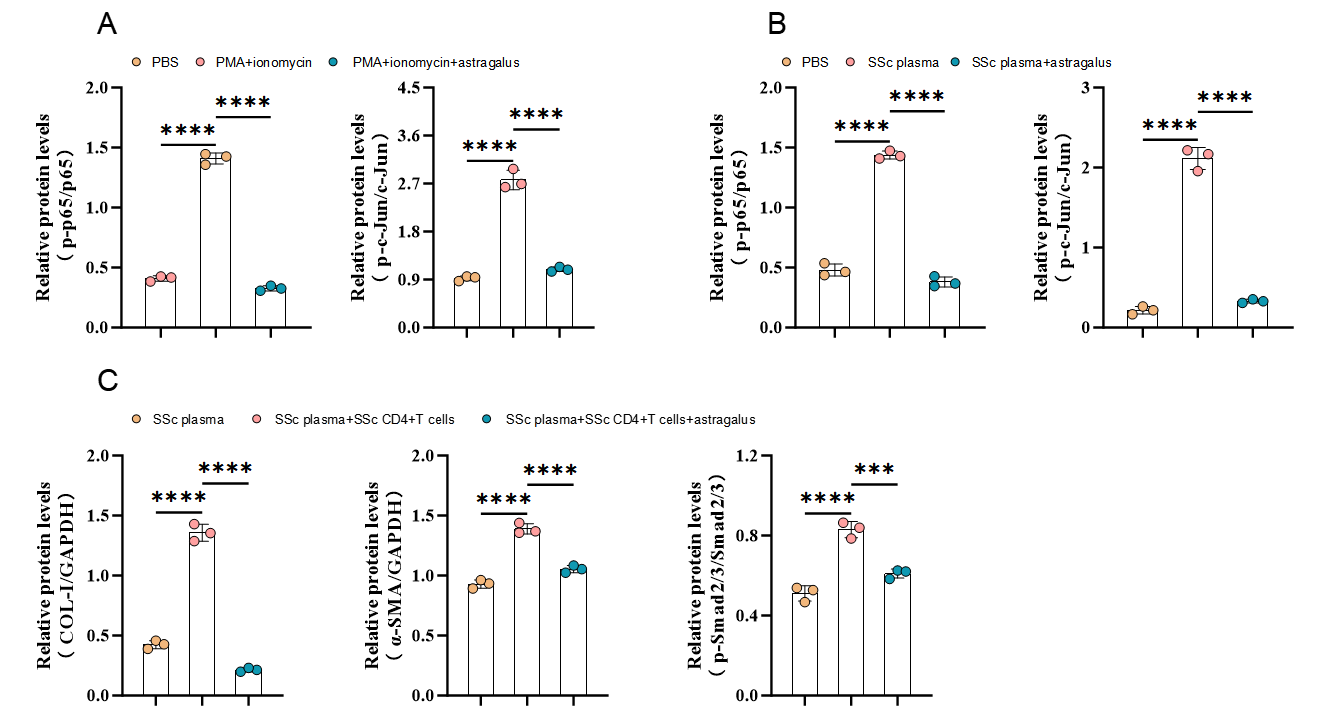


**Supplementary Figure 5. Densitometric quantification of Western blot results from Figure 7.** (A–B) Quantitative analysis of p-p65 and p-c-Jun protein levels in Jurkat T cells (A, from Fig. 7C) and primary CD4+ T cells (B, from Fig. 7F). (C) Quantification of COL-I, α-SMA, and p-Smad2/3 expression in SSc dermal fibroblasts (from Fig. 7H). Data are mean ± SD; p < 0.01, *** p < 0.001, **** p < 0.0001 (one-way ANOVA).


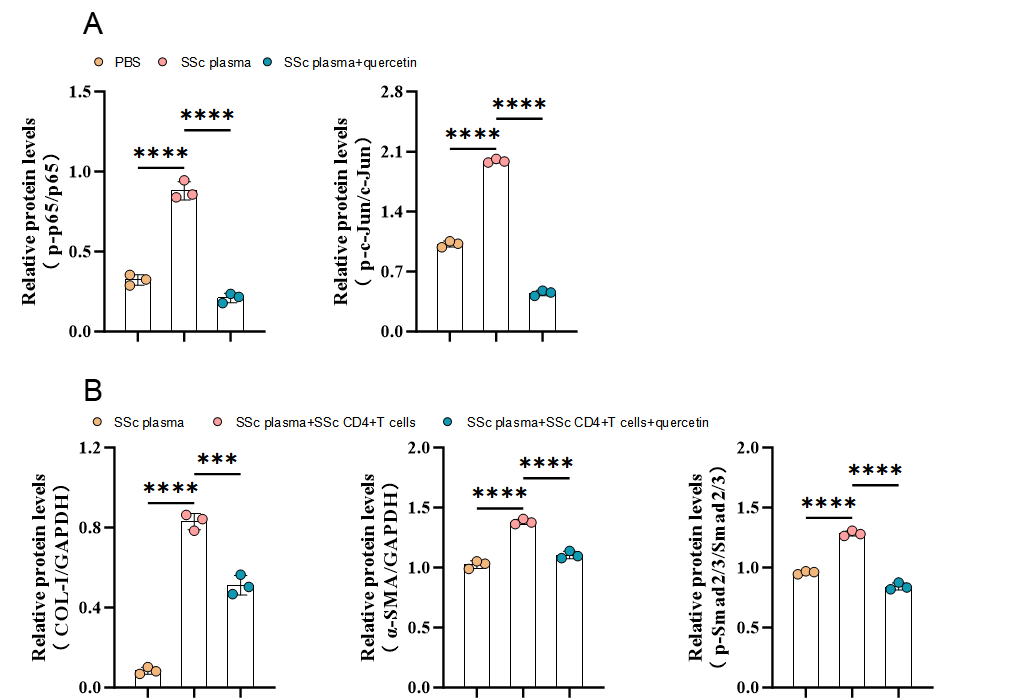


**Supplementary Figure 6.** Densitometric quantification of Western blot results from Figure 8. (A) Quantitative analysis of p-p65 and p-c-Jun protein levels in SSc CD4+ T cells (from Fig. 8C). (B) Quantification of COL-I, α-SMA, and p-Smad2/3 expression in SSc dermal fibroblasts (from Fig. 8E). Data are mean ± SD; p < 0.01, *** p < 0.001, **** p < 0.0001 (one-way ANOVA).


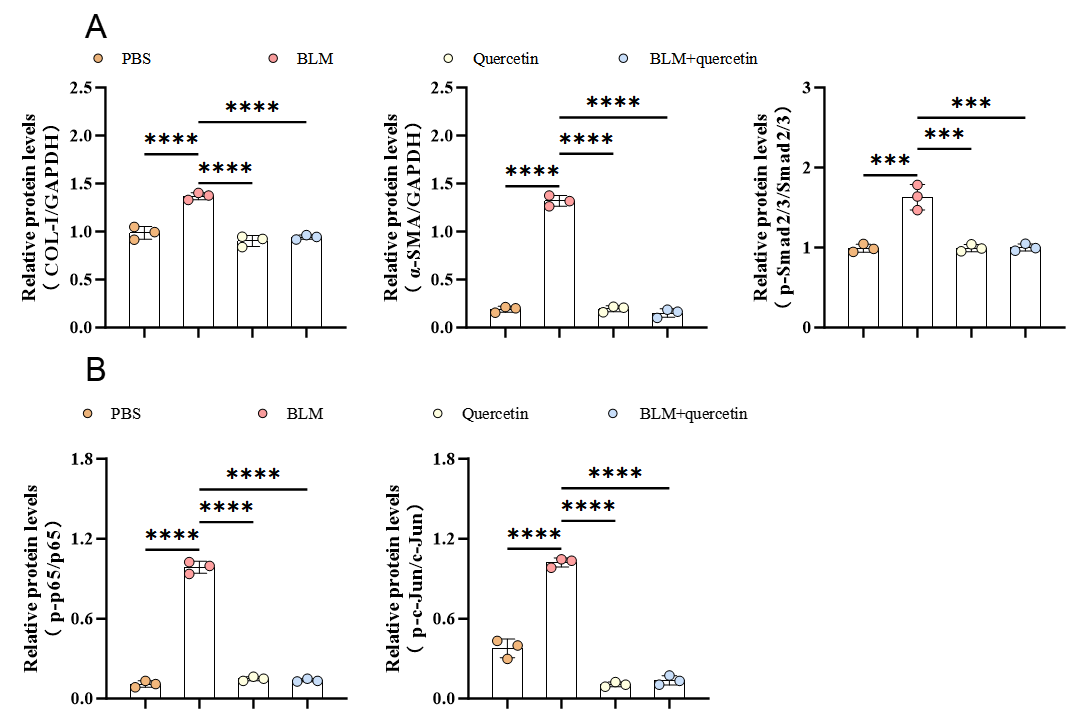


**Supplementary Figure 7.** Densitometric quantification of Western blot results from Figure 9. (A) Quantitative analysis of COL-I, α-SMA, and p-Smad2/3 expression in mouse skin tissues (from Fig. 9J). (B) Quantification of p-p65 and p-c-Jun protein levels in splenic CD4+ T cells (from Fig. 9K). Data are mean ± SD; p < 0.01, *** p < 0.001, **** p < 0.0001 (one-way ANOVA).
