# Supplementary material for: Quercetin attenuates skin inflammation and fibrosis in systemic sclerosis by targeting the RELA/c-Jun axis to suppress th17 cell responses
Source: Front Immunol. 2026 Jun 3;17:1863530. doi: 10.3389/fimmu.2026.1863530 (PMC13272162; doi:10.3389/fimmu.2026.1863530)
Supplement: Supplementary file 2 [file Table2.docx]

**Supplementary Materials and Methods**

1. **Chemical Candidates and Compound**

Selection Chemical candidates relevant to SSc were derived from Astragalus. Active compounds were retrieved from the Traditional Chinese Medicine Systems Pharmacology Database and Analysis Platform (TCMSP) (https://old.tcmsp-e.com/tcmsp.php). Screening criteria included oral bioavailability (OB) > 30% and drug-likeness (DL) > 0.18. A total of 20 active compounds with favorable pharmacokinetic profiles were identified for further investigation.

1. **Construction of the Protein–Protein Interaction Network and Identification of Hub Genes**

A protein–protein interaction (PPI) network was constructed from common targets using the STRING database (https://string-db.org/). The network was visualized and analyzed with Cytoscape v3.10.1. Hub genes were identified based on degree values, calculated using the CytoHubba plugin in Cytoscape, with genes exhibiting degree values ≥ 60 selected for downstream analysis.

1. **T Cell Culture and Treatment**

Jurkat T cells and CD4+T cells isolated from SSc patient peripheral blood were cultured in RPMI-1640 complete medium. For Jurkat T cells treated with Astragalus, cells were activated with PMA and ionomycin prior to treatment. Both cell supernatants and pellets were collected for subsequent analysis. The protein levels of inflammatory cytokines in the supernatants were measured by ELISA, while the transcriptional levels of target genes in the cells were determined by qPCR. For CD4+T cells directly treated with Astragalus, both cell supernatants and pellets were similarly collected. The inflammatory cytokine levels in the supernatants were analyzed by ELISA, and the transcriptional levels of relevant genes in the cells were assessed by qPCR.

1. **Construction of the Protein–Protein Interaction Network and Identification of Hub Genes**

A protein–protein interaction (PPI) network was constructed from common targets using the STRING database (https://string-db.org/). The network was visualized and analyzed with Cytoscape v3.10.1. Hub genes were identified based on degree values, calculated using the CytoHubba plugin in Cytoscape, with genes exhibiting degree values ≥ 60 selected for downstream analysis.

1. **Functional Enrichment and Pathway Analysis**

Functional enrichment was performed on the potential therapeutic targets and hub genes using Gene Ontology (GO) and Kyoto Encyclopedia of Genes and Genomes (KEGG) pathway analysis. The analyses were conducted in RStudio (R version 4.3.0) using “org.Hs.eg.db,” “clusterProfiler,” “GOplot,” and “enrichplot” packages. GO terms and KEGG pathways with adjusted p-values < 0.05 were considered significant.

1. **CCK-8 cell viability assay**

Jurkat T cells were maintained in RPMI-1640 medium supplemented with 10% fetal bovine serum (FBS). A stock solution of Astragalus extract was prepared by dissolving granules in distilled water (5 g/mL) and subsequently diluted in complete medium to yield final working concentrations ranging from 100 ng/mL to 5 mg/mL (specifically 5 mg/mL, 2 mg/mL, 1 mg/mL, 750 μg/mL, 500 μg/mL, 250 μg/mL, 1 μg/mL, and 100 ng/mL). Similarly, quercetin was prepared at final working concentrations of 1, 2, 4, 8, 16, 32, and 64 μM. Cells were seeded into 96-well plates at a density of 5 × 10³ cells/well in 200 μL of complete medium containing the indicated concentrations of *Astragalus* or quercetin. The plates were incubated for 24, 48, and 72 h. Four hours prior to each measurement time point, the culture medium was gently replaced with 200 μL of fresh complete medium, followed by the addition of 20 μL of CCK-8 reagent per well. Following an additional 4-h incubation period, the optical absorbance was measured at 450 nm using a microplate reader. Cell viability was calculated relative to the optical density values of the untreated control wells.

1. **Immunohistochemistry (IHC) for mouse skin tissue**

Paraffin-embedded skin sections were deparaffinized in two changes of xylene (10 min each) and rehydrated through a descending graded ethanol series (100%, 100%, 95%, 80%, and 60%; 5 min per step), followed by three washes in deionized water (3 min each). Heat-induced epitope retrieval was performed in a microwave using an appropriate antigen retrieval buffer, after which the sections were cooled and rinsed with deionized water (3 × 1 min). A hydrophobic barrier was delineated around the tissue sections using a PAP pen, and non-specific binding was prevented by incubating the sections with a rapid blocking buffer for 20 min at room temperature; excess buffer was gently removed without washing. The sections were then incubated overnight at 4°C with the following primary antibodies: anti-mouse IL-17A, IL-4, IFN-γ, p-p65, p-Jun, and p-Fos. Following three washes in Tris-buffered saline (TBS; 1 min each), the sections were incubated with 50 μL of horseradish peroxidase (HRP)-conjugated secondary polymer (anti-rabbit/mouse) for 30 min at room temperature, and subsequently washed again with TBS (3 × 1 min). Colorimetric detection was achieved by applying 50 μL of 3,3’-diaminobenzidine (DAB) substrate; the chromogenic reaction was monitored microscopically until an optimal brown signal developed (typically 2–3 min) and was immediately terminated by rinsing with deionized water (3 × 1 min). Where applicable, signal enhancement was conducted using 1% CuSO₄ for 5 min, followed by rinsing in deionized water (3 × 1 min). Nuclei were counterstained with hematoxylin for 2–3 min, rinsed in TBS, blued in TBS buffer for 5 min, and washed in deionized water (3 × 1 min). The sections were dehydrated through an ascending graded ethanol series (60% to 100%; 5 min per step) and cleared in two changes of xylene (5 min each). Finally, the slides were mounted with coverslips using a neutral mounting medium and imaged using a brightfield microscope.

1. **Immunofluorescence (IF) staining for mouse skin tissue**

Slides were horizontally positioned in staining racks and baked at 65°C for 20 min, followed by natural air drying at room temperature. The sections were deparaffinized and rehydrated by sequential immersion in an eco-friendly dewaxing solution I (10 min), dewaxing solution II (10 min), absolute ethanol (4 min), 95% ethanol (4 min), 85% ethanol (4 min), 75% ethanol (4 min), 50% ethanol (4 min), and ultimately double-distilled H₂O (ddH₂O). Slides were subsequently submerged in a prepared antigen retrieval solution. The retrieval solution was pre-heated in a microwave at 100% power for 3 min until boiling. The slides were transferred into the boiling retrieval solution and subjected to a customized microwave heating cycle: 100% power for 30 s to boiling, 20% power for 4 min, 20% power for an additional 8 min, and a final 100% power pulse for 30 s until boiling. The staining box was then removed and allowed to cool naturally to room temperature. The sections were washed with an immunostaining wash buffer on an orbital shaker. Excess liquid was gently tapped off, and residual moisture surrounding the tissue was carefully removed using lint-free paper, ensuring the sample areas were not touched. A hydrophobic pen was used to demarcate the tissue sample regions. A rapid blocking buffer was applied to each defined zone, and the slides were incubated at 37°C for 20 min. Excess blocking buffer was decanted, and 30 μL of primary antibody (anti-α-SMA) was applied to the respective tissue zones. The slides were incubated horizontally in a humidified chamber at 37°C for 1.5 h. Following primary antibody incubation, the slides were washed three times with TBST on a shaker (5 min per wash). Fluorophore-conjugated secondary antibody was applied, and the slides were incubated in a humidified, light-protected chamber at 37°C for 2 h. Excess secondary antibody was removed, and the slides were subjected to a brief soak in TBST, followed by four 10-min washes on a shaker. The sections were mounted using an antifade mounting medium with coverslips, sealed with nail polish, and stored in the dark at 4°C for 1 h prior to imaging.

1. **Enzyme-Linked Immunosorbent Assay (ELISA)**

Protein levels of IL-17A, IFN-γ, and IL-4 were quantified using specific ELISA kits obtained from Lianke Biotech (Catalog Nos. EK217, EK280, and EK204, respectively). Assays were performed using pre-coated 96-well microplates; briefly, 100 μL of cell culture supernatant or mouse plasma was added to each well along with 5 μL of detection antibody. Plates were sealed and incubated at room temperature for 2 h on an orbital shaker. Following incubation, plates were washed six times with the provided wash buffer. Subsequently, 100 μL of horseradish peroxidase (HRP)-conjugated streptavidin was added, and plates were incubated for an additional 30 min at room temperature with shaking. After six additional washes, 100 μL of TMB substrate was added, and the reaction was allowed to proceed in the dark for 30 min at room temperature. The colorimetric reaction was terminated by adding 100 μL of stop solution per well. Absorbance was recorded at 450 nm and 570 nm using a microplate reader

1. **Histological staining (H&E and Masson’s trichrome)**

For H&E staining, paraffin-embedded sections were deparaffinized in xylene (or an eco-friendly alternative) and rehydrated through a graded alcohol series to water. Tissues were stained with high-definition hematoxylin for 3 min, differentiated, blued, and subsequently counterstained with eosin Y for 20 s. Sections were then rapidly dehydrated through absolute ethanol, cleared in xylene, and mounted with neutral balsam.

For Masson’s trichrome staining, sections were processed using a commercial kit (BioBioPha, Cat# BBL-0200). Deparaffinized sections were fixed in Bouin's solution at 37°C for 1 h, stained with Weigert’s iron hematoxylin for 5 min, followed by Ponceau red staining for 10 min. After phosphomolybdic acid differentiation for 15 min, sections were counterstained with aniline blue, dehydrated, and mounted for microscopic evaluation.

1. **Quantification of dermal hydroxyproline content**

Dermal hydroxyproline levels were quantified utilizing a Hydroxyproline Assay Kit (Elabscience, Cat# E-BC-K062-M). Skin tissue (100 mg) was minced and hydrolyzed in 1 mL of 6 M HCl at 95°C for 6 h. The hydrolysate pH was neutralized (pH 6.5–7.0) using the provided buffer solutions and brought to a final volume of 10 mL with double-distilled water. The mixture was treated with Reagent IX (20 mg) and centrifuged at 1,500 × g for 10 min. The supernatant (400 μL) or standard was reacted with Reagent I working solution for 5 min, followed by Reagent IV working solution at 60°C for 15 min. Absorbance was measured at 558 nm using a microplate reader, and hydroxyproline content (per 100 mg tissue) was extrapolated from a standard curve.
