# Supplementary material for: Quercetin attenuates skin inflammation and fibrosis in systemic sclerosis by targeting the RELA/c-Jun axis to suppress th17 cell responses
Source: Front Immunol. 2026 Jun 3;17:1863530. doi: 10.3389/fimmu.2026.1863530 (PMC13272162; doi:10.3389/fimmu.2026.1863530)
Supplement: Supplementary file 3 [file Table3.docx]

**Supplementary table 1 Primer sequences**

| **Human gene** | **Primer sequences** |
| --- | --- |
| IL-17A | Forward Primer: 5’-TCCCACGAAATCCAGGATGC -3’ |
|  | Reverse Primer: 5’-GGATGTTCAGGTTGACCATCAC -3’ |
| IFN-γ | Forward Primer: 5’-TCGGTAACTGACTTGAATGTCCA -3’ |
|  | Reverse Primer: 5’-TCGCTTCCCTGTTTTAGCTGC -3’ |
| IL-4 | Forward Primer: 5’-GTTGCTGCTTGCAGTAACCTT-3’ |
|  | Reverse Primer: 5’-AGGGCCAAGTCCAACTCCTT-3’ |
